# The Configuration of RPA, RAD51, and DMC1 Binding in Meiosis Reveals the Nature of Critical Recombination Intermediates

**DOI:** 10.1016/j.molcel.2020.06.015

**Published:** 2020-08-20

**Authors:** Anjali Gupta Hinch, Philipp W. Becker, Tao Li, Daniela Moralli, Gang Zhang, Clare Bycroft, Catherine Green, Scott Keeney, Qinghua Shi, Benjamin Davies, Peter Donnelly

**Affiliations:** 1 Wellcome Centre for Human Genetics, University of Oxford, Oxford, UK; 2 Howard Hughes Medical Institute, Molecular Biology Program, Memorial Sloan Kettering Cancer Center, New York, NY 10065, USA; 3 Hefei National Laboratory for Physical Sciences at the Microscale, The CAS Key Laboratory of Innate Immunity and Chronic Diseases, School of Life Sciences, University of Science and Technology of China, Hefei, Anhui, China; 4 Department of Statistics, University of Oxford, Oxford, UK

**Keywords:** recombination, meiosis, RAD51, RPA, DMC1, DNA double-strand breaks, DNA repair, strand invasion, D-loop, crossover

## Abstract

Meiotic recombination proceeds via binding of RPA, RAD51, and DMC1 to single-stranded DNA (ssDNA) substrates created after formation of programmed DNA double-strand breaks. Here we report high-resolution *in vivo* maps of RPA and RAD51 in meiosis, mapping their binding locations and lifespans to individual homologous chromosomes using a genetically engineered hybrid mouse. Together with high-resolution microscopy and DMC1 binding maps, we show that DMC1 and RAD51 have distinct spatial localization on ssDNA: DMC1 binds near the break site, and RAD51 binds away from it. We characterize inter-homolog recombination intermediates bound by RPA *in vivo*, with properties expected for the critical displacement loop (D-loop) intermediates. These data support the hypothesis that DMC1, not RAD51, performs strand exchange in mammalian meiosis. RPA-bound D-loops can be resolved as crossovers or non-crossovers, but crossover-destined D-loops may have longer lifespans. D-loops resemble crossover gene conversions in size, but their extent is similar in both repair pathways.

## Highlights

• DMC1 binds near and RAD51 away from DNA break sites on homology search filaments
• RPA binds the repair template chromosome in mammalian meiotic D-loops *in vivo*
• DMC1, not RAD51, performs strand exchange in mammalian meiotic DNA break repair
• D-loops resemble crossover gene conversion tracts in size and localization

## Graphical Abstract

**Figure:**
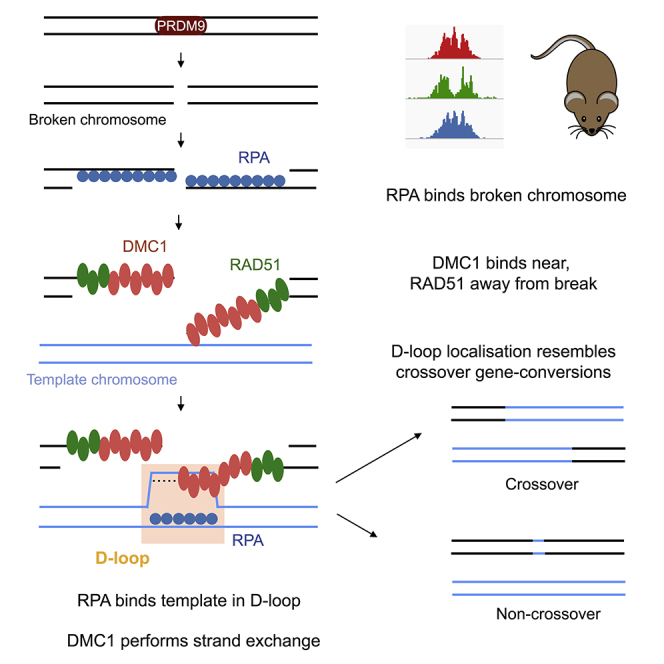

## Teaser

Hinch et al. show that DMC1 and RAD51 bind substrates created after formation of DNA breaks with distinct localization in mouse meiosis. DMC1 binds near break sites and performs strand exchange with the homologous chromosome. RPA binds the template chromosome in D-loop intermediates, which resemble crossover gene conversions in size and localization, in multiple repair pathways.

## Introduction

Recombination is a vital process for creation of gametes in sexually reproducing species and is required for correct segregation of homologous chromosomes during meiosis (Cole et al., 2012a). Recombination, together with mutation, generates all genetic variation and creates the substrate for evolution via natural selection.

**Figure 1:**
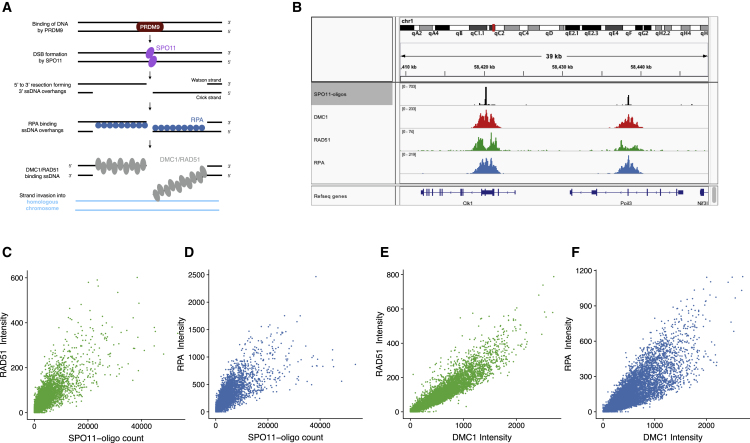
Genome-wide Maps of RAD51, RPA, and DMC1 Binding in Meiosis (A) PRDM9 binds DNA at particular sequence motifs, some of which become sites of breaks by SPO11. 5′-to-3′ strand resection occurs, creating ssDNA overhangs, which are bound first by RPA (blue) and then by RAD51 and DMC1 (shown in gray to indicate that their relative localization is unknown). Our ChIP-seq maps measure complexes of these proteins with ssDNA (but not dsDNA). (B) An Integrative Genomics Viewer illustration of SPO11 oligos (measuring DSBs, black), DMC1 (red), RAD51 (green), and RPA (blue) ssDNA ChIP-seq reads in B6 in a segment of chromosome 1 with two hotspots ~18 kb apart. (C and D) Comparison of measures of RAD51 and RPA binding (C and D, respectively), with the number of SPO11 oligos in autosomal hotspots identified using DMC1 (n = 16,926) in B6. The intensity of RAD51 and RPA binding is a strand-aware and background-adjusted measurement of the number of ChIP-seq reads. (E and F) Comparison of measures of RAD51 and RPA binding (E and F, respectively), with DMC1 binding (Hinch et al., 2019) in autosomal hotspots (n = 23,631) in the hybrid.

In many species, meiotic recombination clusters into narrow regions of the genome, called “hotspots” (Baudat et al., 2013). An early step in recombination in many vertebrates is binding of DNA by the histone methyltransferase PRDM9 in a sequence-specific manner (Baudat et al., 2013; Figure 1A). A few hundred PRDM9-bound sites are subject to formation of DNA double-strand breaks (DSBs) by the protein SPO11 (Lange et al., 2016). The broken DNA then performs a search for the homologous chromosome to serve as a template for repair. For a small number of breaks, this results in formation of crossovers, whereas the majority repair without a crossover.

After formation of a DSB, DNA ends are resected in the 5′-to-3′ direction to produce 3′ single-stranded DNA (ssDNA) overhangs (Figure 1A). These are thought to be bound initially by the ssDNA binding protein RPA (Brown and Bishop, 2014; Crickard and Greene, 2018). RPA is thought to be replaced in part or fully by the mammalian RecA orthologs DMC1 and RAD51, which bind the overhangs to form nucleoprotein filaments (Figure 1A). The filaments search for the matching sequence on the homologous chromosome and carry out strand invasion (Crickard and Greene, 2018). DMC1 is expressed only during meiosis, whereas RAD51 is expressed in meiotic and non-meiotic cells (Brown and Bishop, 2014). RAD51 is necessary for repair of DSBs by homologous recombination in non-meiotic cells, with its absence being embryonic lethal (Lim and Hasty, 1996; Tsuzuki et al., 1996). In meiosis, DMC1 is essential for recombination in mammals and many other species, but the meiotic function of RAD51 remains unclear, especially in mammals (Bannister and Schimenti, 2004; Dai et al., 2017). RAD51 performs strand exchange in meiosis in *Caenorhabditis elegans*, which lacks DMC1 (Woglar and Villeneuve, 2018), and also in *Schizosaccharomyces pombe* (Murayama et al., 2008) in combination with DMC1. RAD51 is necessary for meiosis in *Saccharomyces cerevisiae* and *Arabidopsis thaliana* (Cloud et al., 2012; Da Ines et al., 2013), but its ability to perform strand exchange is dispensable (Bishop, 2012; Cloud et al., 2012; Da Ines et al., 2013; Singh et al., 2017). Instead, it has a role in promoting the formation and function of DMC1 filaments (Bishop, 2012; Chan et al., 2019). In the absence of DMC1, RAD51 can perform strand exchange and effect DSB repair in certain *S. cerevisiae* mutants and *A. thaliana*, but chromosome segregation and crossover formation remain defective (Da Ines et al., 2012; Lao et al., 2013). The precise localization of DMC1 and RAD51 on ssDNA filaments also remains unclear. Although a study in *S. cerevisiae* has indicated that RAD51 and DMC1 localize side by side on resected DSB ends (Brown et al., 2015), little co-localization was observed in *A. thaliana* (Kurzbauer et al., 2012).

RPA is the major eukaryotic ssDNA binding protein and is essential for life, with functions in DNA replication and repair (Liu and Huang, 2016). It displays extremely high affinity for ssDNA; indeed, the presence of RPA is interpreted as evidence of the existence of ssDNA (Kim et al., 1992). RPA foci are present early in meiosis when DSBs are forming (Pacheco et al., 2018). This is consistent with a role of RPA in binding nascent ssDNA overhangs preceding and promoting formation of RAD51- and DMC1-bound nucleoprotein filaments (Shi et al., 2019; Soustelle et al., 2002). However, numerous RPA foci are also present later in meiosis, when autosomes have synapsed and new DSBs are not expected to be made (Moens et al., 2002; Oliver-Bonet et al., 2007; Pacheco et al., 2018; Woglar and Villeneuve, 2018). This suggests an additional role of RPA in recombination after formation of nucleoprotein filaments but before formation of crossovers (Chan et al., 2019; Shi et al., 2019). The nature of this role, however, remains unclear. It is also not clear whether the roles of RPA differ between somatic cells (Wang and Haber, 2004) and meiotic cells, as observed for other proteins, particularly given the existence of meiosis-specific proteins (MEIOB and SPATA22) resembling subunits of RPA (Ribeiro et al., 2016; Xu et al., 2017).

There continues to be limited understanding, especially regarding mammals, of the process of meiotic recombination in the stages between creation of DSBs and formation of crossovers (Hinch et al., 2019; Hunter and Kleckner, 2001; Oh et al., 2007), in large part because of the dynamic nature of the reactions and the transience of potential intermediates. Here we map the binding positions of RAD51 and RPA genome-wide in the mouse with high sensitivity, specificity, and spatial resolution. Together with the equivalent DMC1 data and cytological localization of these proteins, these maps answer long-standing questions about the functions and localization of RAD51, RPA, and DMC1 in meiosis, the nature of inter-homolog recombination intermediates, and the dynamics of DSB repair.

## Results

### Genome-wide Maps of RAD51 and RPA Binding in Meiosis

We performed chromatin immunoprecipitation followed by ssDNA sequencing (ChIP-seq) (Khil et al., 2012) for RAD51 and RPA (specifically RPA2, which forms the RPA complex with RPA1 and RPA3; Liu and Huang, 2016) in testes of adult male wild-type *Mus musculus domesticus* C57BL/6J (hereafter called B6) and a hybrid strain (Hinch et al., 2019). These assays, together with the published DMC1 assays in these mice (Brick et al., 2012; Hinch et al., 2019), specifically enrich for and are informative about proteins bound to ssDNA (but not double-stranded DNA [dsDNA]). The B6 mouse is homozygous for a wild-type *Prdm9* allele (*Prdm9*^B6^). The hybrid is a fertile cross between a genetically modified B6 mother homozygous for an engineered *Prdm9* allele harboring a DNA-binding domain found in many human populations (*Prdm9*^HUM^) (Davies et al., 2016) and a *Mus musculus castaneus* CAST/EiJ (hereafter called CAST) father homozygous for the wild-type CAST *Prdm9* allele (*Prdm9*^CAST^). Hereafter, we refer to this mouse as B6CASTF1^HUM/CAST^ or simply “the hybrid.” The hybrid is a particularly powerful model because sequence differences between pairs of homologous chromosomes often allow us to localize events specifically to one or the other of the two homologs. We also chose these mice for the rich set of stage-specific data already available, including DSB locations in B6 (Lange et al., 2016), DMC1 maps in B6 and the hybrid (Brick et al., 2012; Hinch et al., 2019), and crossover and non-crossover locations in the hybrid (Hinch et al., 2019; Li et al., 2019), which enable us to link early steps in recombination through to their final outcomes.

We found that RAD51 and RPA ChIP-seq reads cluster into small regions that, in B6, match known recombination hotspots identified by mapping of DSB locations via SPO11 oligos (Lange et al., 2016) (Figure 1B). We measured the signal of RAD51 and RPA in previously identified DMC1 hotspots. The intensity of RAD51 and RPA correlated strongly with SPO11 oligos in them (Figures 1C and 1D; Pearson’s correlation coefficient [*r*] = 0.85 and *r* = 0.84, respectively, for autosomal hotspots) in B6. They also correlated strongly with DMC1 in B6 (Figures S1A and S1B; *r* = 0.94 and *r* = 0.91, respectively) and in the hybrid (Figures 1E and 1F; *r* = 0.95 and *r* = 0.89, respectively). We also identified 6,677 and 9,039 peaks of RAD51 and 9491 and 10,482 peaks of RPA *de novo* (B6 and the hybrid, respectively), the vast majority of which overlapped DMC1 hotspots. We conclude that the assays for RPA and RAD51 measure their presence sensitively and accurately at recombination hotspots.

### RAD51 and DMC1 Bind ssDNA on the DSB-Initiating Chromosome Only, whereas RPA Binds the DSB-Initiating and Repair Template Chromosomes

**Figure 2:**
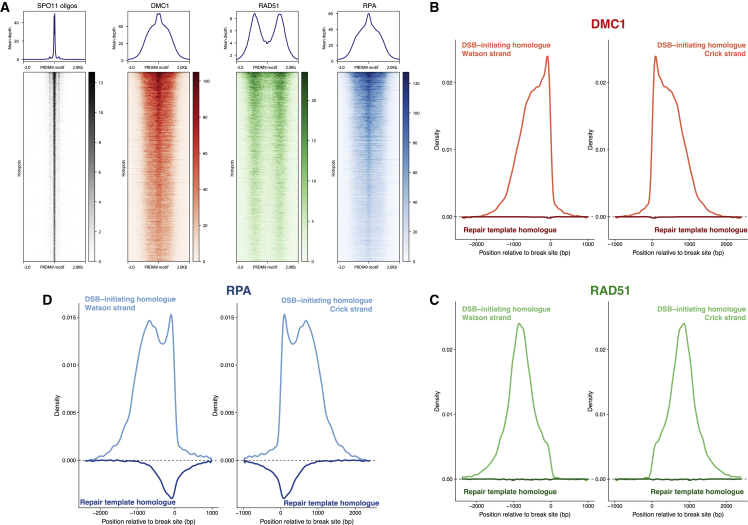
RAD51 and DMC1 Bind ssDNA on the DSB-Initiating Chromosome with Distinct Localization, whereas RPA Binds the DSB-Initiating and Repair Template Chromosomes (A) Heatmaps showing coverage of SPO11 oligos (black), DMC1 (red), RAD51 (green), and RPA (blue) ChIP-seq reads in hotspots identified by DMC1 ChIP-seq in B6. Each row represents one of the 4,000 most active hotspots with a well-defined PRDM9-binding motif, ordered by the number of SPO11 oligos. The coverage shown is centered relative to the midpoint of each PRDM9-binding site. The numerical coverage values are dependent on experimental factors (e.g., sequencing depth) and are not comparable between experiments. (B) Density of DMC1 binding on the DSB-initiating (light red) and repair template (dark red) homologs relative to DSB sites, separated for Watson (left) and Crick (right) strands. Density was inferred in asymmetric PRDM9^CAST^ hotspots with a well-defined PRDM9-binding motif in the hybrid (n = 1,955) via deconvolution and normalized so that the area under the curves for each strand is 1 (100-bp smoothing). (C) As in (B) but for RAD51 binding on the DSB-initiating (light green) and repair template (dark green) homologs, separated for Watson (left) and Crick (right) strands. (D) As in (B) but for RPA binding on the DSB-initiating (light blue) and repair template (dark blue) homologs, separated for Watson (left) and Crick (right) strands.

The fine-scale anatomy of recombination hotspots has remained elusive, and it is currently not known where RPA and RAD51 bind within a hotspot. RPA has not been measured previously in meiosis beyond the resolution of foci visible in cytological assays, whereas RAD51 has been assayed only in an infertile mutant with severe strand exchange defects (Khil et al., 2012; Smagulova et al., 2011). We compared heatmaps of SPO11 oligos (Lange et al., 2016), DMC1 (Brick et al., 2012), RAD51, and RPA within PRDM9^B6^ hotspots in the B6 mouse (Figure 2A). Although SPO11 oligos, which measure DSBs, are concentrated within a few hundred bases around the PRDM9-binding motif, DMC1 has a wide footprint, with the highest intensity close to the hotspot center. In contrast, RAD51 is concentrated in twin peaks flanking hotspot centers. RPA has a strong concentration close to the center of the hotspot and a wide overall footprint. Heatmaps in the hybrid also show a similar localization of these proteins (Figure S2A). In subsequent analyses, we use the statistical technique of deconvolution to calculate binding profiles of these proteins relative to the DSB site by taking into account the distribution of DSBs relative to the hotspot center (Figure S2B; Lange et al., 2016).

Recombination requires the chromosome on which a DSB occurs to engage with its homolog, and key recombination proteins may bind one or both homologs at different times. Our assays measure protein binding averaged over millions of cells, so it is not possible to distinguish their behavior in an individual cell. Nevertheless, in the hybrid mouse, we can identify sites in which DSBs occur on only one of the B6 or CAST homologs by utilizing sequence polymorphisms between them (Davies et al., 2016; Hinch et al., 2019; Keane et al., 2011). These so-called asymmetric hotspots are usually the result of polymorphisms affecting the PRDM9-binding motif, leading to differences in PRDM9 binding between the homologs (Davies et al., 2016; Hinch et al., 2019). Within these asymmetric hotspots, one homolog can thus be identified as being the “DSB-initiating homolog,” and the other as the “repair template homolog.” For the following analyses, we selected hotspots where recombination is, on average, ∼20 times more likely to initiate on one of the two homologs (Davies et al., 2016; Hinch et al., 2019).

The DMC1 ChIP-seq signal is confined to the DSB-initiating chromosome only, as expected from DMC1 binding 3′ ssDNA overhangs associated with programmed DSBs (Figures 2B and S2C). We found that the entirety of the RAD51 signal is also contributed by the DSB-initiating chromosome, with no detectable RAD51-bound ssDNA from the repair template (Figures 2C and S2D). This is consistent with RAD51, like DMC1, binding 3′ ssDNA overhangs following DSB resection. The twin peaks of RAD51 binding (Figure 2A) are on different strands; the peak to the left of the hotspot center represents binding to the Watson strand and the peak to the right on the Crick strand, consistent with the location of ssDNA overhangs after resection (Figure 1A). There are, however, very clear differences between the localization of the two recombinases (Figures 2B and 2C), which we discuss below. (Our assays are designed to detect binding specifically with ssDNA, not dsDNA [Khil et al., 2012]. When strand exchange proteins engage with the repair template, they are bound to dsDNA [Brown and Bishop, 2014], and the assays do not measure that binding.)

In contrast to DMC1 and RAD51, there is substantial RPA ChIP-seq signal on the DSB-initiating and repair template chromosomes (Figures 2D, S2E, and S2F). Further, the localization of RPA on them is distinct. On the DSB-initiating chromosome, RPA extends from the break site to ∼2 kb to the right on the Crick strand (left on the Watson strand). These observations support the current model that RPA binds 3′ ssDNA overhangs created by DSB processing.

In contrast, RPA on the repair template chromosome overlaps the break site for the Watson and Crick strands, with substantial binding on both sides of the break. On the Crick strand, ∼70% of the signal is to the right of the break and ∼30% to the left (conversely for the Watson strand). We analyze below where and when each of these proteins binds the DSB-initiating and repair template chromosomes.

### RAD51 and DMC1 Preferentially Occupy Distinct Parts of ssDNA Overhangs, with DMC1 Occupying the End Closest to the Break Site

The peak in DMC1 coverage on the DSB-initiating chromosome is 80 bp from the break, with the median position 420 bp from it (Figures 2B and S2G). In contrast, the peak and median RAD51 positions are further away, 860 bp and 800 bp from the break, respectively (Figures 2C and S2G). This indicates that, although DMC1 preferentially occupies ssDNA close to the break site, RAD51 typically binds more distally.

To define the spatial disposition of DMC1 relative to RAD51 at individual sites of recombination, we sought a cytological correlate of the ChIP-seq data. Prior studies in *S. cerevisiae* revealed side-by-side localization of Rad51 and Dmc1 complexes, with both proteins apparently on both sides of individual DSBs (Brown et al., 2015). However, in *A. thaliana*, these proteins have been reported to be spatially separated, perhaps on opposite DSB ends (Kurzbauer et al., 2012). In the mouse, co-localization of RAD51 and DMC1 in conventional and electron microscopy was interpreted as the presence of mixed complexes (Tarsounas et al., 1999), but the possibility of a side-by-side arrangement has not been addressed.

**Figure 3:**
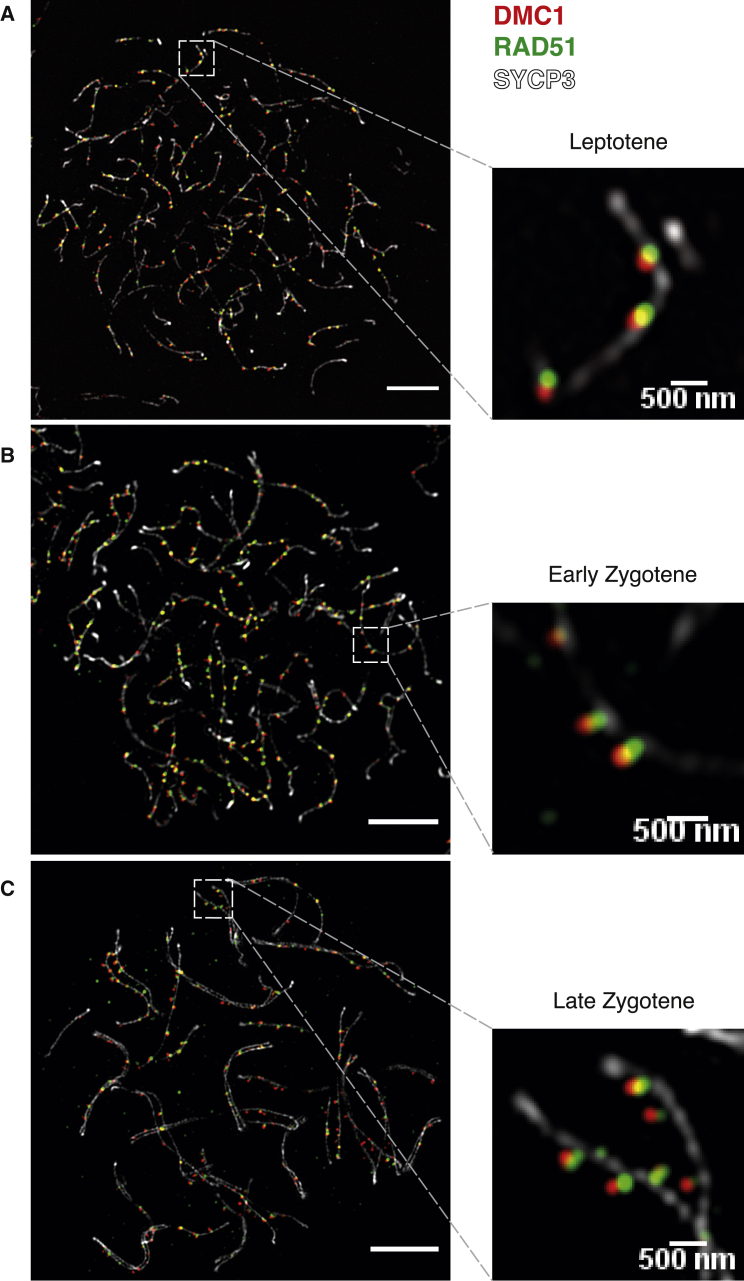
RAD51 and DMC1 Foci Form Paired Co-foci (A–C) Representative images from structured illumination microscopy of spread spermatocytes in different meiotic stages, stained for DMC1 (red), RAD51 (green), and SYCP3 (white) in 13–15 dpp (days postpartum) B6 × DBA/2J mice. SYCP3 is a major component of chromosome axes (Baudat et al., 2013). Scale bars, 5 μm (500 nm for magnified images).

We used high-resolution structured illumination microscopy to define relative locations of RAD51 and DMC1 on chromosome spreads of individual spermatocytes. Consistent with prior analyses by conventional microscopy (Moens et al., 2002; Tarsounas et al., 1999), we observed numerous immunostaining foci of RAD51 and DMC1 (Figures 3A–3C and S3A–S3C). Nearly all DMC1 foci (97%) and the majority of RAD51 foci (81%) were associated with chromosome axes. Among axis-associated foci, most DMC1 and RAD51 foci occurred together in pairs; 82.3% of RAD51 and 81.6% of DMC1 foci were within 300 nm of a focus of the other protein (Figures 3 and S3D). Importantly, however, these foci were nearly always offset from one another, with a median offset of 120 nm (Figure S3D).

This observed offset is at an appropriate size scale for both foci to be in an individual ssDNA overhang on the same side of a DSB (Sheridan et al., 2008). Moreover, these findings agree well with studies in yeast, including the distances between foci (Brown et al., 2015). Therefore, we infer that RAD51 and DMC1 nucleoprotein filaments tend to occur together in pairs and that this side-by-side arrangement at individual DSBs underlies the population-average offset between RAD51 and DMC1 in our ChIP-seq data. Interestingly, we also observed that RAD51 foci tended to be closer to unsynapsed chromosome axes than DMC1 (p = 10^−15^, *t* test; Figures 3, S3E, and S3F). Additional experiments confirmed that neither the offset of RAD51 from DMC1 nor its axis proximity are imaging artifacts (Figures S4 and S5; STAR Methods).

**Figure 4:**
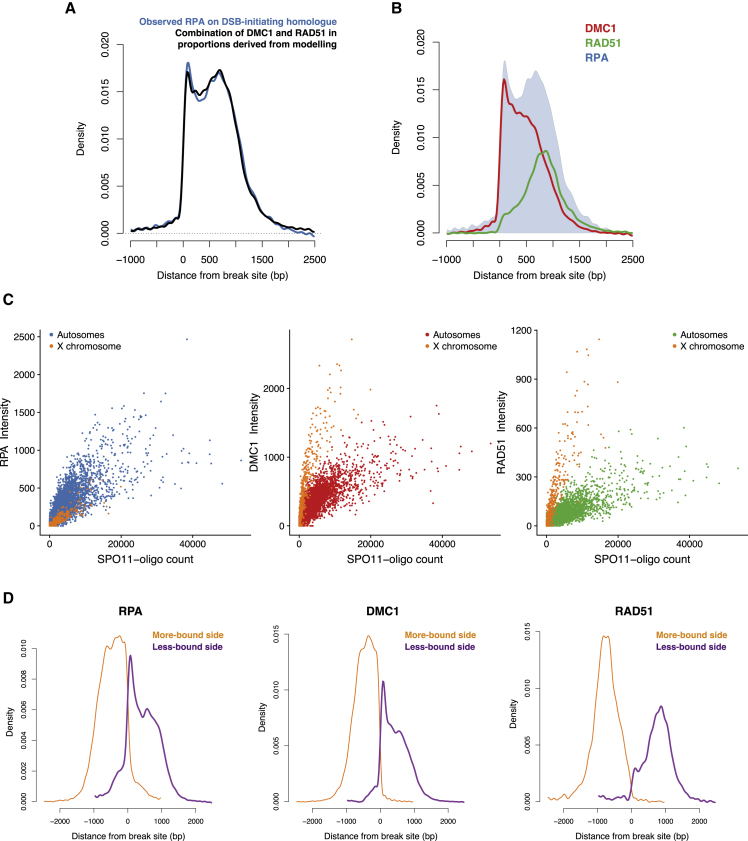
Relative Binding of DMC1, RAD51, and RPA on the DSB-Initiating Homolog (A) Comparison of observed RPA binding on the DSB-initiating homolog (blue) with the best-fitting linear combination of DMC1 and RAD51 binding inferred by modeling (65% and 35%, respectively; the Crick strand is shown; see also Figure S6A). (B) Composition of the ssDNA nucleoprotein filament inferred by modeling (Crick strand). Shown is RPA on the DSB-initiating homolog (light blue background) with inferred proportions of DMC1 (red) and RAD51 (green). RPA is normalized so that the area under its curve is 1, with DMC1 and RAD51 proportions being 0.65 and 0.35, respectively (100-bp smoothing). (C) Comparison of measures of RPA (left), DMC1 (center), and RAD51 (right) binding, with the SPO11-oligo count in autosomal (n = 16,926) and non-pseudoautosomal X chromosome hotspots (n = 1,103, orange) in B6. (D) Density of RPA (left), DMC1 (center), and RAD51 (right) in hotspots where DMC1 binding is greater on one strand (orange) than on the other strand (purple) in B6. Only skewed hotspots with a well-defined PRDM9 motif and over 8 kb away from the nearest hotspot were included (n = 6,340). For purposes of illustration, all skewed hotspots were co-oriented to have greater binding on the left. These plots show protein binding relative to break sites (after deconvolution) and are thus not confounded by DSB localization (see also Figure S7B).

The current model for recombination after DSB formation is that DMC1 and RAD51 replace RPA from nascent ssDNA overhangs. This model is supported by several lines of evidence (Pacheco et al., 2018; Shi et al., 2019; Soustelle et al., 2002) and is compatible with our data. We can ask: what proportions of DMC1 and RAD51 most closely match RPA binding, and do they accurately re-capitulate it? Signals for different proteins cannot be compared quantitatively because they are affected by experimental factors. Therefore, we compared only the shapes of the binding distributions, which are affected by relative differences in binding of a protein to different parts of an ssDNA overhang but not by the total amount of binding or immunoprecipitation. We found that the shape of the RPA binding profile on the DSB-initiating chromosome is reproduced accurately and precisely by a combination of the DMC1 and RAD51 profiles (Figure 4A), which supports the current model. The best-fitting proportions for RPA shape in PRDM9^CAST^ hotspots are 65% DMC1 and 35% RAD51, with similar proportions for PRDM9^HUM^ hotspots (DMC1:RAD51 = 62%:38%). Alternative proportions (for instance, equal proportions) do not recapitulate the shape of RPA distribution (Figure S6A). Assuming that the lifespan of RPA binding is similar across the ssDNA and that RPA does not return to bind the DSB-initiating chromosome after being replaced by DMC1 and RAD51, this implies that there is nearly twice as much DMC1 as RAD51 on ssDNA filaments on average. Note that this is the time-averaged occupancy inferred over millions of cells and not necessarily the instantaneous configuration at any particular time. The relative occupancy of ssDNA strands is summarized in Figure 4B. We cannot exclude the possibility that RPA returns to bind the DSB-initiating chromosome at a later stage; e.g., after strand exchange. However, if that were to occur, then our data place substantial constraints on possible models (STAR Methods).

It has remained unclear whether RPA remains present on the nucleoprotein filaments after DMC1 and RAD51 have loaded (Brown et al., 2015; Shi et al., 2019). To address this question, we exploited systematic differences between hotspots; namely, that the DMC1 signal is elevated relative to DSB frequency in hotspots where breaks take longer to engage with their homologs (Hinch et al., 2019). A well-known example is breaks on the non-pseudoautosomal region of the X chromosome, which lacks a homolog in males (Davies et al., 2016; Lu and Yu, 2015). We observed a comparable strong elevation in RAD51 and DMC1 signals in these hotspots but no elevation whatsoever in the RPA signal (Figures 4C and S6B–S6E). For autosomal hotspots where search for the homolog takes longer (Hinch et al., 2019), we again observed a strong elevation in the RAD51 signal but no consistent differences in the RPA signal (Figures S6F–S6H). This implies that RPA is not present extensively on ssDNA filaments along with DMC1 and RAD51 during homology search. Similarly, these data argue strongly against models in which RPA is bound to ssDNA on one side of a DSB, whereas DMC1 and RAD51 are bound to the other side during homology search. However, these data are compatible with transient co-occupancy of RPA with DMC1 or RAD51; e.g., during the loading of RAD51 and DMC1 on ssDNA (Shi et al., 2019).

We performed further analyses to understand the shape of RPA binding on the DSB-initiating chromosome (Figure 2D). We observed that numerous hotspots in B6 and the hybrid display a skewed pattern of protein binding (43% and 40%, respectively), with more of the RPA, DMC1, and RAD51 signals on one side of a DSB than the other (Figures 4D and S7A). Among these hotspots, similar numbers had more binding on the Watson as on the Crick strand (p = 0.27, binomial test). In addition to differences in the total amount, the shape of protein binding, particularly of RPA and DMC1, are strikingly different on the more-bound and less-bound sides (Figure 4D). This difference cannot be explained by a skew in the location of DSBs (Figures 4D and S7B). Importantly, it is correlated with downstream consequences (Figures S7C–S7E), with a greater concentration of non-crossovers (Table S1; p = 5 × 10^−7^, binomial test; Figure S7C) and crossover breakpoints (Table S2; p = 2 × 10^−4^, binomial test) on the more-bound side. The localization of non-crossovers suggests an increase in gene conversions unusually distant from the break site on the more-bound side (Figures S7C–S7E). There is also more RPA on the repair template on the more-bound side (Figure S8A). These clear downstream consequences in recombination outcomes establishes that the skewed binding pattern is not an artifact (Figures S7–S9; STAR Methods). Further analyses suggest that the imbalance may be driven by differences in the efficiency of break processing and protein loading on the two sides (Figure S8B), with the skew in DMC1 and RAD51 binding potentially a consequence of a skew in RPA loading (Figure S8C). Recent work has indicated that incomplete processing of one of the two DSB ends occurs often in the mouse (Paiano et al., 2020); however, it remains to be investigated whether our observations reflect related phenomena.

### RPA Binds the Repair Template Chromosome within Inter-homolog Recombination Intermediates, which May Be Resolved as Crossovers or Non-crossovers

**Figure 5:**
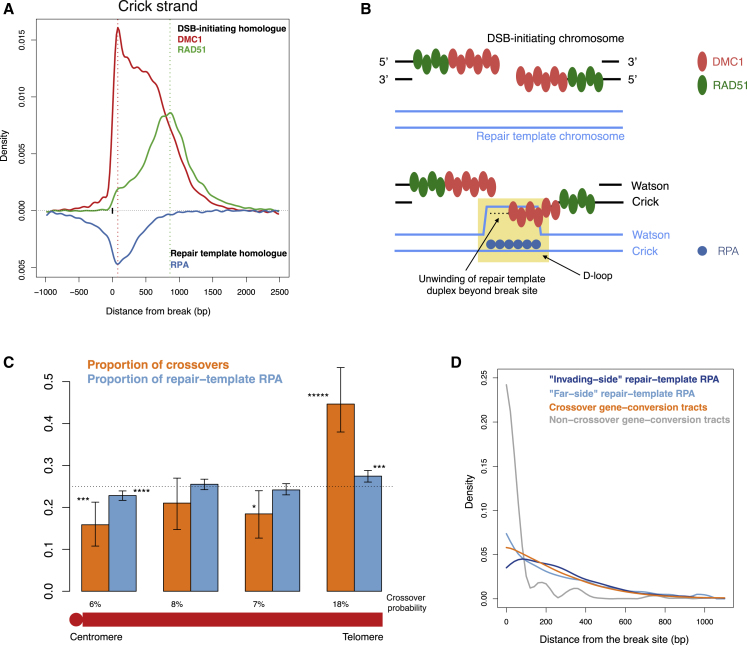
RPA Binds the Repair Template Chromosome within Inter-homolog Recombination Intermediates, which May Be Resolved as Crossovers or Non-crossovers (A) Comparison of RPA (blue) on the repair template homolog, with DMC1 (red) and RAD51 (green) on the DSB-initiating homolog, relative to the break site (solid black tick). Dotted lines indicate positions of peak DMC1 (red) and RAD51 (green) binding (only the Crick strand is shown for clarity). (B) Model of RPA binding on the repair template chromosome. DMC1 (red) and RAD51 (green) bind ssDNA overhangs on the DSB-initiating chromosome (black), with DMC1 close to the DSB and RAD51 nearer the junction with dsDNA. A segment of the DMC1-bound nucleoprotein filament invades and pairs with the repair template chromosome (light blue). The corresponding strand of the repair template chromosome becomes single stranded (known as the displacement loop or D-loop, highlighted in yellow) and is bound by RPA (dark blue). The D-loop is extended, potentially after further unwinding of the DNA duplex and binding of RPA ahead of the DNA polymerase. (C) Comparison of crossover resolution probability and RPA signal on the repair template chromosome between hotspots in different regions of chromosomes (for the same estimated number of DSBs). Asymmetric hotspots in the hybrid (n = 4,218) were divided into 4 bins with an equal RPA signal on the DSB-initiating chromosome, based on distance to the centromere. The proportion of crossovers (orange) and RPA on the repair template (blue) is shown for each bin. The proportion expected from the number of DSBs in each bin is 0.25 (dotted black line). Error bars show 95% confidence intervals estimated using 100,000 bootstrap iterations. The number of asterisks indicates the significance (n asterisks indicate p < 10^−n^). The numbers below the bars indicate the estimated fraction of inter-homolog recombination intermediates that are repaired as crossovers (STAR Methods). (D) Comparison of RPA binding on the repair template chromosome on the same side as the invading strand (dark blue; i.e., to the right of the break site on the Crick strand and left of it on the Watson strand), RPA on the other side of the break site (light blue), with the probability of a site being within crossover-associated (orange) and non-crossover-associated (gray) gene conversion tracts. Curves were normalized to have area 1 under each curve to facilitate comparison.

Our ability to map RPA binding to individual homologous chromosomes (Figure 2D) not only establishes a role of RPA on the repair template in the process of homolog engagement, but it allows us to characterize its extent. Most of the signal for RPA on the repair template chromosome ranges from −560 bp to 740 bp relative to the break site (the central 95% of the signal; Figures 5A and S10A). This establishes the existence of an inter-homolog recombination intermediate in which at least one of the two strands of the repair template chromosome becomes single-stranded close to and overlapping the site corresponding to the break site on the DSB-initiating chromosome.

Specifically, the RPA signal on the repair template chromosome (Figure 5A) points to the displacement loop (D-loop) (Figure 5B), a long-hypothesized and much-explored recombination intermediate (Eggler et al., 2002; Hunter and Kleckner, 2001; Sneeden et al., 2013; Sugiyama et al., 1997; Wang and Haber, 2004) whose properties *in vivo* remain unknown. In this model, the single-stranded DMC1 and/or RAD51 nucleoprotein filament on the DSB-initiating chromosome invades the repair template chromosome and engages with one of its strands via Watson-Crick pairing (Figure 5B). As a result, the other strand of the repair template chromosome becomes single stranded and is thought to be stabilized by RPA (Chan et al., 2019; Eggler et al., 2002; Sneeden et al., 2013). This model is consistent with findings from electron micrography in mouse spermatocytes that show that most RPA foci co-localize with DMC1/RAD51 foci when the autosomes are synapsing in zygotene (Moens et al., 2002). RPA foci appear to lose DMC1 and RAD51 over the course of pachytene (Moens et al., 2002), which is consistent with the D-loop persisting after DMC1 and RAD51 removal. Strand invasion is followed by extension of the D-loop, possibly after further unwinding of the DNA duplex and binding of RPA ahead of the DNA polymerase (Sneeden et al., 2013).

Interestingly, the signal for RPA binding is greater on the side of the break site that is homologous to the invading filament than on the other side (>2-fold difference; Figure 5A). This may be explained by persistence of the initial D-loop prior to its extension. A not mutually exclusive possibility is that part of the signal comes from RPA binding to the template strand in addition to the displaced strand of the repair template chromosome (Sneeden et al., 2013). Our data permit us to define spatial and temporal characteristics of meiotic D-loops *in vivo*, as detailed below.

RPA on the repair template chromosome overlaps strongly with DMC1 on the DSB-initiating chromosome (Figure 5A). The peak in DMC1 signal on the DSB-initiating chromosome matches the peak in RPA signal on the repair template chromosome. In contrast, RAD51 tapers off sharply in regions of high RPA binding on the repair template (Figure 5A). In fact, the points at which RAD51 peaks or at which RAD51 binding exceeds DMC1 binding correspond to very low levels of RPA on the template (Figure 5A). The segment of the DSB-initiating chromosome that engages in strand invasion displaces the homologous sequence on the repair template (Figure 5B); therefore, the presence of RPA corresponding to high levels of DMC1, but not RAD51, specifically implicates DMC1 in the process of strand invasion. Together with the independent lines of evidence above, these data provide *in vivo* support for a model in which only DMC1, but not RAD51, performs strand exchange in mammalian meiosis. This is consistent with findings in *S. cerevisiae* and *A. thaliana* (Bishop, 2012; Cloud et al., 2012; Da Ines et al., 2013).

A small fraction of DSBs in the mouse are resolved as crossovers (∼10%), with the majority being repaired as non-crossovers (Cole et al., 2012a; 2012b). In *S. cerevisiae*, the model for the decision between crossover and non-crossover repair pathways posits that differentiation occurs at an early stage, before stable strand exchange and chromosome synapsis (Bishop and Zickler, 2004), whereas data in some other organisms, including the mouse, motivate progressive implementation of the decision (Cole et al., 2012a; de Boer et al., 2006; Qiao et al., 2014; Rao et al., 2017). Previous cytological work suggests that intermediates detected as RPA foci in zygotene and pachytene may be resolved as crossovers or non-crossovers (de Boer et al., 2006; Moens et al., 2002; Oliver-Bonet et al., 2005; 2007; Woglar and Villeneuve, 2018; Figures S10B and S10C). Hence, it is natural to ask whether the RPA-bound intermediates we detect differ, in lifespan or in localization, when the crossover outcome is more or less likely. To investigate this, we exploited known systematic differences between hotspots in the likelihood of a break being repaired as a crossover or a non-crossover. Mouse chromosomes are acrocentric, and DSBs in hotspots close to the non-centromeric telomere in males are far more likely to be repaired as crossovers than DSBs elsewhere (Brick et al., 2018; de Boer et al., 2006; Hinch et al., 2019). Therefore, we compared the proportion of crossovers and the signal for RPA on the repair template in hotspots with the same expected number of breaks but in different parts of chromosomes (Figures 5C, S10D, and S10E). We observed that hotspots near the telomere, which have higher crossover resolution (p < 10^−5^), also have a significantly higher proportion of RPA on the repair template (p = 3 × 10^−4^; Figure 5C). Correspondingly, hotspots near the centromere have a significantly lower proportion of both crossovers (p = 7 × 10^−4^) and repair template RPA (p = 9 × 10^−5^; Figure 5C) per break. This establishes a positive association between crossover outcome and RPA on the repair template chromosome (p = 10^−5^), which, in turn, implies that the RPA-bound intermediates cannot be exclusive to the non-crossover pathway. On the other hand, the magnitude of the difference (Figures 5C, S10D, and S10E) is inconsistent with these intermediates occurring only on the crossover pathway (STAR Methods). This implies that RPA-bound intermediates exist within both repair pathways, with crossover-destined intermediates having a longer lifespan or greater amount of DNA bound to RPA or both. We examine these possibilities below.

To assess whether the localization and size of RPA-bound intermediates vary for different repair outcomes, we again compared hotspots between chromosomal regions with large differences in crossover resolution. We found no difference in RPA localization between them (Figure S10F). We also did not observe a difference between chromosomes that have more or fewer MLH1 foci per DSB (p = 0.46) (Froenicke et al., 2002). A parsimonious interpretation of these findings is that RPA-bound intermediates are similar in size and shape within crossover and non-crossover pathways but have a longer lifespan *en route* to crossovers. Under reasonable assumptions, the average lifespan of intermediates that are ultimately resolved as crossovers is 2- to 3-fold greater than those resolved as non-crossovers. We cannot rule out the possibility that intermediates destined to become non-crossovers could be individually smaller but more dispersed, balancing out precisely in a population of cells to match the localization of crossover-destined intermediates. However, non-crossovers observed in this hybrid are not dispersed in this manner, instead being strongly concentrated near breaks (Li et al., 2019; see below). We therefore favor the longer-lifespan model, which is also more parsimonious.

Next we compared the localization of RPA-bound intermediates with that of gene conversion tracts associated with crossovers and non-crossovers. We leveraged crossovers identified previously at high resolution in the hybrid (Hinch et al., 2019) (n = 1,021) to estimate the gene conversion tracts associated with them. Specifically, we found that the probability that a given site is within a crossover gene conversion tract is comparable with the probability of having RPA bound to it on either side of the break (Figure 5D). This is consistent with the most parsimonious model, where the extent of DNA re-synthesis using the homolog as a template is similar to the extent to which the homolog duplex is unwound during crossover repair. In contrast, non-crossover tracts are much shorter (Cole et al., 2014; Li et al., 2019) and closer to break sites than RPA binding (Figure 5D). This suggests that, despite having RPA-bound intermediates of comparable size, DNA polymerization associated with non-crossovers is limited by other means.

## Discussion

It has proven difficult to obtain direct evidence of the steps in meiotic recombination between formation of programmed DSBs and the endpoints of DSB repair as crossovers or non-crossovers. Here we characterized the binding locations of the ssDNA binding proteins RPA2, RAD51, and DMC1 at high resolution genome-wide in meiosis in B6 and genetically modified B6CASTF1 hybrid mice. Crucially, the hybrid allows separate study of protein binding on the chromosomes on which DSBs take place compared with those that are used as templates for repair. This has enabled novel mechanistic insights into the intervening steps in recombination.

We show that RPA, DMC1, and RAD51 each bind the resected ssDNA produced after DSB formation, with RPA likely not present at appreciable levels with DMC1 and RAD51 during homology search. The binding locations within hotspots genome-wide and our high-resolution micrographs within individual cells show that DMC1 is loaded preferentially at the cut end of ssDNA overhangs, with RAD51 loaded preferentially at the opposite end. Our modeling suggests that more DMC1 than RAD51 is present on the filaments on average. This implies that previous estimates of ssDNA resection based solely on DMC1 binding (Lange et al., 2016) are underestimates, in agreement with recent work (Paiano et al., 2020; Yamada et al., 2020).

Our observation of extensive RPA binding on the repair template chromosome establishes the existence of a long-lived recombination intermediate that has the properties and structure hypothesized for the D-loop (Brown and Bishop, 2014; Hunter, 2015). We show that, averaged across cells, hotspots, and time, the D-loop extends on both sides of the DSB (−560 to +740 bp). We also show that many DSBs that are repaired as non-crossovers spend time, albeit less so, in the same intermediate as those that are resolved as crossovers, an intermediate that is similar in size to crossover-associated gene conversion tracts.

The extent of RPA binding on the repair template is contained within the DMC1 signal on the DSB-initiating chromosome, and the binding patterns of these proteins have matching peaks. In contrast, sites of high RAD51 binding correspond to near-zero levels of RPA on the repair template. Together with the evidence that DMC1 is located preferentially at the cut end of the ssDNA filament, these data support a model in which DMC1, but not RAD51, performs the function of strand exchange in mammalian meiosis. This is consistent with findings in *S. cerevisiae* and *A. thaliana* and does not exclude alternative, possibly accessory roles of RAD51 (Bishop, 2012; Cloud et al., 2012; Da Ines et al., 2013; Lao et al., 2013). These data lend weight to the interpretation that robustness of meiosis to loss of RAD51 reflects that RAD51 is not directly involved in strand exchange, as opposed to other explanations, such as compensation by DMC1.

We propose a model that combines our data and the existing body of evidence (reviewed in Baudat et al., 2013; Brown and Bishop, 2014; Hunter, 2015). RPA, which binds nascent ssDNA overhangs created by strand resection, is replaced by DMC1 nearer the DSB and RAD51 nearer the junction with dsDNA. A segment of the DMC1 filament closest to the break site invades the homolog, displacing a repair template strand in a D-loop recombination intermediate. Subsequently, the displaced strand of the repair-template chromosome becomes bound by RPA. Cytological and ChIP-seq data of RPA are perhaps most parsimoniously explained by a model in which a substantial proportion of or all breaks proceed via the same RPA-bound intermediate, with differentiation between the repair pathways occurring later. In the event that there are more inter-homolog intermediates than necessary for crossovers, a subset is repaired as non-crossovers (Hinch et al., 2019; Woglar and Villeneuve, 2018). This model predicts a progression in the degree of interference observed between RPA foci, with little interference at the outset of synapsis, and an increasing degree as the number of foci declines over the course of pachytene (de Boer et al., 2006; Oliver-Bonet et al., 2007). For DSBs that are repaired with a crossover, the intermediate is completed by DNA polymerization and double Holliday junction formation. The similarity between the physical extent of RPA-bound intermediates and crossover gene conversion tracts supports this model. Endpoints of crossover gene conversion tracts are modulated by the chromatin environment on the repair template (Hinch et al., 2019). This suggests that extension of the D-loop is influenced by the presence of nucleosomes on the template chromosome. For DSBs that are repaired as non-crossovers, another mechanism must ensure that DNA synthesis from the homolog is limited; for instance, via disruption of the D-loop followed by synthesis-dependent strand annealing (Brown and Bishop, 2014).

Use of ChIP-seq data has certain limitations. Signals from our assays are derived from millions of cells and represent averages across time through meiosis. Any intermediates that are present over timescales that are substantially shorter than those for which we see a signal are invisible to us. Independent sources of information, such as cytological assays, are necessary to identify the sequence of binding events. For instance, it is possible that some of the RPA binding we observe on the DSB-initiating chromosome occurs after stable strand exchange, when DMC1 and RAD51 have been removed but before re-synthesis of the resected DNA. The signal from RPA bound to any inter-sister recombination intermediates cannot be distinguished from RPA bound to the chromatid on which the DSB occurs, and it is possible that part of the signal on the DSB-initiating chromosome is due to repair with the sister chromatid. However, the excellent fit of the RPA signal to a combination of DMC1 and RAD51 (Figure 4A) indicates that any such RPA signal because of inter-sister recombination on the autosomes is small. This suggests that strand exchange with the sister chromatid in autosomal hotspots occurs infrequently, is short lived, or both (Oh et al., 2007).

Recombination during meiosis is a highly choreographed process involving dozens, if not hundreds, of proteins acting at different stages (Jung et al., 2019). Joint analysis of the trio of high-resolution, time-sensitive genome-wide RPA, RAD51, and DMC1 assays provides a powerful new toolkit for analysis of subtle changes and catastrophic disruptions to the meiotic program.

## STAR★Methods

### Key Resources Table

**Table:** 

REAGENT or RESOURCE	SOURCE	IDENTIFIER
**Antibodies**		

Mouse monoclonal anti-RPA2 antibody (RPA34-20)	Calbiochem	Cat # NA19L, RRID:AB_565123
Mouse monoclonal anti-Rad51 antibody (14B4)	Novus Biologicals	Cat # NB100-148, RRID:AB_10002131
Dynabeads® M-280 Sheep Anti-Mouse IgG	Invitrogen	Cat# 11201D, RRID:AB_2783640
Mouse monoclonal anti-SYCP3	Abcam	Cat# ab97672, RRID:AB_10678841
Rabbit polyclonal anti-DMC1(H-100)	Santa Cruz Biotechnology	Cat# sc-22768, RRID:AB_2277191
Rabbit polyclonal anti-RAD51(H-92)	Santa Cruz Biotechnology	Cat# sc-8349, RRID:AB_2253533
Guinea pig polyclonal anti-DMC1	This study	N/A
Donkey anti-guinea pig IgG H&L (FITC)	Jackson ImmunoResearch Labs	Cat# 706-095-148, RRID:AB_2340453
Donkey anti-mouse IgG H&L (Alexa fluor 488)	Thermo Fisher Scientific	Cat# A-21202, RRID:AB_141607
Donkey anti-rabbit IgG H&L (Alexa fluor 488)	Thermo Fisher Scientific	Cat# A-21206, RRID:AB_2535792
Donkey anti-rabbit IgG H&L (Alexa fluor 555)	Thermo Fisher Scientific	Cat# A-31572; RRID: AB_162543
Goat anti-guinea pig IgG H&L (Alexa fluor 488)	Thermo Fisher Scientific	Cat# A-11073, RRID:AB_2534117
Goat anti-guinea pig IgG H&L (Alexa Fluor 555)	Thermo Fisher Scientific	Cat# A-21435, RRID:AB_2535856
Goat anti-guinea pig IgG H&L (Alexa Fluor 594)	Thermo Fisher Scientific	Cat# A-11076, RRID:AB_141930
Goat anti-mouse IgG H&L (CF405S)	Biotium	Cat# 20080-1, RRID:AB_10852977
Goat anti-mouse IgG H&L (Alexa Fluor 647)	Thermo Fisher Scientific	Cat# A-21235, RRID:AB_2535804
Mouse monoclonal anti-DMC1 clone 2H12/4	Abcam	Cat# ab11054, RRID:AB_297706
Mouse polyclonal anti-RAD51	Abcam	Cat# ab88572, RRID:AB_2042762
Rabbit polyclonal anti-RPA2	Abcam	Cat# ab10359, RRID:AB_297095
Biotinylated rabbit polyclonal anti-SYCP3	Novus Biologicals	Cat# NB300-232, RRID:AB_2087193
Rabbit polyclonal anti-SYCP3	Abcam	Cat# ab15093, RRID:AB_301639
Mouse polyclonal anti-SYCP3 clone D1	Santa Cruz Biotechnology	Cat# sc-74569, RRID:AB_2197353
Streptavidin Cy5	ThermoFisher Scientific	Cat# SA1011
Goat anti-rabbit Alexa Fluor 488	ThermoFisher Scientific	Cat# A-11034, RRID:AB_2576217
Goat anti-rabbit Alexa Fluor 594	ThermoFisher Scientific	Cat# A-11012, RRID:AB_141359
Donkey anti-mouse Alexa Fluor 488	ThermoFisher Scientific	Cat# A-32766, RRID:AB_2762823
Donkey anti-mouse Alexa Fluor 594	ThermoFisher Scientific	Cat# A-32744, RRID:AB_2762826

**Deposited Data**		

Raw and processed RPA and RAD51 ChIP-seq data	This study	GEO: GSE143582
Structured Illumination Microscopy data for RAD51 and DMC1	This study	Mendeley: https://doi.org/10.17632/gwwk2jnr82.1
SPO11-oligo data	Lange et al., 2016	GEO: GSE84689
DMC1 ChIP-seq data for B6 (data were remapped to mm10)	Brick et al., 2012	GEO: GSM869781 and GSM869782
DMC1 ChIP-seq data for hybrid	Hinch et al., 2019	GEO: GSE124991
Crossover locations in hybrid	Hinch et al., 2019	GEO: GSE125326
Non-crossover locations in hybrid	Li et al., 2019	https://idp.nature.com/authorize?response_type=cookie&client_id=grover&redirect_uri=https%3A%2F%2Fwww.nature.com%2Farticles%2Fs41467-019-11675-y

**Experimental Models: Organisms/Strains**		

Mouse: C57BL/6J	Charles River, The Jackson Laboratory	RRID:IMSR_JAX:000664
Mouse: CAST/EiJ	MRC Harwell	Stock code: MCAM, RRID:IMSR_JAX:000928
Mouse: DBA/2J (used to generate mice with a mixed DBA2/B6 background)	The Jackson Laboratory	Strain#: 000671, RRID:IMSR_JAX:000671
Mouse: Prdm9^tm1.1(PRDM9)Wthg^	Davies et al., 2016	MGI: 5708566
Mouse: *Spo11* knockout	Baudat et al., 2000	N/A
Mouse: *Dmc1* knockout	Pittman et al., 1998	N/A

**Software and Algorithms**		

ChIP-seq reads mapping and hotspot calling	Brick et al., 2012; Davies et al., 2016	https://github.com/anjali-hinch/hybrid-rescue
Deeptools	Ramírez et al., 2016	https://deeptools.readthedocs.io/en/develop/
R	N/A	http://www.r-project.org
Motif calling	Altemose et al., 2017	https://elifesciences.org/articles/28383
NIS-Elements software	Nikon	N/A
Fiji	Schindelin et al., 2012	https://imagej.net/Welcome

### Resource Availability

#### Lead Contact

Further information and requests for resources and reagents should be directed to and will be fulfilled by the Lead Contact, Peter Donnelly (peter.donnelly@genomicsplc.com)

#### Materials Availability

The genetically manipulated humanized model, Prdm9^tm1.1(PRDM9)Wthg^, is available from the authors on request.

#### Data and Code Availability

The raw and processed sequencing files produced in this study are archived at the NCBI Gene Expression Omnibus (GEO; https://www.ncbi.nlm.nih.gov/geo/) under accession number GEO: GSE143582. Microscopy data are available from Mendeley Data under Mendeley Data: https://doi.org/10.17632/gwwk2jnr82.1. Code has been published previously (Davies et al., 2016; Hinch et al., 2019) and is available from GitHub (https://github.com/anjali-hinch/hybrid-rescue).

### Experimental Model and Subject Details

#### Mice

The breeding of the hybrid mouse was carried out in accordance with UK Home Office Animal [Scientific Procedures] Act 1986, with procedures reviewed by the Clinical Medicine Animal Welfare and Ethical Review Body at the University of Oxford, and conducted under project license PPL 30/3437. Animals were housed in individually ventilated cages, provided with food and water *ad libitum* and maintained on a 12h light:12h dark cycle (150–200 lux). The only reported positives on FELASA health screening over the entire time course of these studies were for *Helicobacter hepaticus* and *Entamoeba spp.* The care and breeding of mice and all animal experiments in China were conducted according to the guidelines of and approved by the Institutional Animal Care Committee of the University of Science and Technology of China. The care and use of mice in the USA were performed in accordance with the Memorial Sloan Kettering Cancer Center (MSKCC) Institutional Animal Care and Use Committee (IACUC). Animals were fed regular rodent chow with *ad libitum* access to food and water.

### Method Details

#### RPA and RAD51 ChIP-seq maps

Chromatin Immunoprecipitation (ChIP) followed by single-stranded DNA sequencing (SSDS) was performed as previously described (Khil et al., 2012) with some modifications: For ChIP, 50μl Dynabeads (M-280 Sheep Anti-Mouse) were washed 3 times with 0.5% BSA in PBS and incubated with 5μg Rad51 Antibody (14B4, Novus) or RPA Antibody (RPA34-20, Calbiochem) in 1ml 0.5% BSA in PBS for at least two hours to bind antibody to beads. Prior to addition of chromatin mixture (see below) beads were washed twice in 0.5% BSA in PBS and resuspended in 100μl 0.5% BSA in PBS.

A detunicated, fresh or fresh-frozen testis of 10 week-old mice was dropped in room temperature 1% formaldehyde (Pierce) in PBS and immediately homogenized in a Dounce homogenizer with 20 strokes of the tight-fit pestle. The homogenate was passed through a 70 μm cell strainer, and after a total of 10 min, fixation was stopped by adding 2.5M glycine to a final concentration of 125mM, followed by 5 min incubation at room temperature. Cells were then collected by centrifugation at 900 g, and washed once with cold PBS. The cell pellet was resuspended in cell lysis buffer (0.25% Triton X-100, 10mM EDTA, 0.5mM EGTA, 10mM Tris-HCl pH = 8.0), incubated for 10 minutes on ice, and centrifuged for 3 minutes at 300 g to collect nuclei. Nuclei were washed in lysis wash buffer (200mM NaCl, 1mM EDTA, 0.5mM EGTA, 10mM Tris-HCl pH = 8.0) and centrifuged 3 min at 300 g. The nuclei pellet was resuspended in 900 μl shearing buffer (0.1% SDS, 10mM Tris-HCl pH = 8.0, 1mM EDTA) supplemented with proteinase inhibitor, split into two ∼500μl aliquots and sonicated at 4°C on a Bioruptor sonicator 15 times for 15 s with 45 s breaks (“low” setting). Sheared nuclei were centrifuged at full speed in a mini centrifuge at 4°C to remove debris. For RPA ChIP, 850μl of chromatin-containing supernatant were mixed with 80μl 5M NaCl, 70μl TE buffer (1mM EDTA, 10mM Tris-HCl pH = 8.0), and 300μl Ren IP buffer (1% Triton X-100, 0.1% Sodium Deoxycholate, 1mM EDTA, 10mM Tris-HCl pH = 8.0). For RAD51 ChIP, 850μl of chromatin-containing supernatant were mixed with 110μl 5M NaCl, 40μl TE buffer (1mM EDTA, 10mM Tris-HCl pH = 8.0), and 300μl Ren IP buffer (1% Triton X-100, 0.1% Sodium Deoxycholate, 1mM EDTA, 10mM Tris-HCl pH = 8.0). This mixture was added to the 100μl Dynabeads bound to either RPA or RAD51 antibody (see above) and incubated overnight on rotating wheel at 4°C for immunoprecipitation. Beads were washed once for 5 minutes each in wash buffer 1 (0.1% SDS, 1% Triton X-100, 2mM EDTA, 20mM Tris-HCl pH = 8.0, 150mM NaCl), wash buffer 2 (0.1% SDS, 1% Triton X-100, 2mM EDTA, 20mM Tris-HCl pH = 8.0, 500mM NaCl), and wash buffer 3 (0.25M LiCl, 1% IGEPAL-CA630, 1% Sodium Deoxycholate, 1mM EDTA, 10mM Tris-HCl pH = 8.0), followed by two brief washes with TE buffer (all washes at 4°C). Elution was performed by incubating the beads in elution buffer (0.1M NaHCO_3_ and 1% SDS) for 30 minutes at 65°C with occasional vortexing, followed by reverse crosslinking of the supernatant at 65°C overnight. To neutralize NaHCO_3_, 4 μl 1M Tris HCl (ph = 6.5) and 2μl 0.5M EDTA were added, after which any RNA was digested by adding 2μl 10mg/ml RNase A for 15 min at 37°C, and proteins were digested by adding 2μl 10mg/ml Proteinase K for 1 hour at 55°C. DNA was purified using ChIP DNA Clean & Concentrator (Zymo Research) using 7 volumes of binding buffer to increase recovery of ssDNA.

SSDS was performed as described previously (Khil et al., 2012). Following end repair and dA-tailing, DNA was incubated at 95°C for 3 minutes and cooled to room temperature to enrich for single-stranded DNA. After ligation with sequencing adaptors, SSDS library was amplified during 12 cycles of PCR. The amplified library was purified, and sequenced on HiSeq2500 using 50bp PE rapid run. The assays were performed once each for RPA and RAD51 on the hybrid and for RPA on the B6 wild-type. For the RAD51 assay on the B6 wild-type, data were pooled from two runs.

We ran a bioinformatic pipeline on the resulting reads for identification of single-stranded sequences (Khil et al., 2012). Hotspot centers were called *de novo* using our published peak-calling algorithm (Davies et al., 2016). We compared the overlap of these hotspots with DMC1 hotspots identified in a variety of mice with different PRDM9 alleles, as previously described (Hinch et al., 2019). The PRDM9 allele of each of these hotspots was also previously identified (Hinch et al., 2019). To assess the agreement of the localization and intensity of peaks of RAD51 and RPA identified *de novo* with DMC1 hotspots, we calculated the total intensity of peaks in each of these classes: peaks overlapping hotspots for each PRDM9 allele, PRDM9-independent hotspots, hotspots where the PRDM9 allele could not be identified, and peaks that did not overlap previously identified hotspots. In the hybrid mouse, the PRDM9 alleles are PRDM^CAST^ and PRDM9^HUM^. The proportion of the signal in RPA peaks identified *de novo* in the hybrid in each of the classes described above are: 66% (PRDM^CAST^), 28% (PRDM9^HUM^), 1% (PRDM9-independent), 5% (PRDM9 allele not identified), 0% (hotspots not overlapping previously identified DMC1 hotspots). The proportion of the signal in RAD51 peaks identified *de novo* in the hybrid in each of the classes described above are: 66% (PRDM^CAST^), 27% (PRDM9^HUM^), 2% (PRDM9-independent), 5% (PRDM9 allele not identified), 0% (hotspots not overlapping previously identified DMC1 hotspots). The proportion of the signal in DMC1 peaks identified *de novo* in the hybrid in each of the classes described above are: 65% (PRDM^CAST^), 30% (PRDM9^HUM^), 0% (PRDM9-independent), 5% (PRDM9 allele not identified), 0% (hotspots not overlapping previously identified DMC1 hotspots). These data show that the RPA and RAD51 signal in this mouse overlap consistently with the DMC1 signal. In the B6 wild-type mouse, the PRDM9 allele is PRDM^B6^ (see main text). The proportion of the signal in RPA peaks identified *de novo* in the wild-type in each of the classes is: 97% (PRDM^B6^), 1% (PRDM9-independent), 2% (PRDM9 allele not identified), 0% (hotspots not overlapping previously identified DMC1 hotspots). The proportion of the signal in RAD51 peaks identified *de novo* in the wild-type in each of the classes is: 80% (PRDM^B6^), 8% (PRDM9-independent), 2% (PRDM9 allele not identified), 11% (hotspots not overlapping previously identified DMC1 hotspots). The proportion of the signal in DMC1 peaks identified *de novo* in the wild-type in each of the classes is: 98% (PRDM^B6^), 0% (PRDM9-independent), 1% (PRDM9 allele not identified), 0% (hotspots not overlapping previously identified DMC1 hotspots). These data show once again that essentially all of the RPA signal in this mouse overlaps corresponding DMC1 hotspots. Although the signal-to-noise ratio of the RAD51 assay on the B6 mouse is somewhat lower than the other assays, we note that the data quality is nevertheless high as evidenced by the high correlation of RAD51 signal with both DMC1 signal (Pearson’s correlation *r* = 0.94) and with SPO11 oligos (Pearson’s correlation *r* = 0.85) in DMC1 hotspots.

In order to have a standardized set of hotspots across which to perform analyses for proteins in this study (DMC1, RAD51 and RPA), we did the following: First, we took previously published DMC1 hotspot sets in these mice (Davies et al., 2016) for B6 and (Hinch et al., 2019) for the hybrid and identified the best matching PRDM9 motif in them (Altemose et al., 2017). The midpoint of the PRDM9 motif was assigned to be its center in the instances where a unique high-quality binding motif could be identified (probability of match > 0.90). A unique high-quality motif match was found for 99.9% of all PRDM9^B6^ and 94.7% of all PRDM9^CAST^ hotspots. Second, we estimated the intensities of DMC1, RPA and RAD51 ChIP-seq using the same, previously published, algorithm (Davies et al., 2016), but with new parameters (hotspot width = 500 bp, binding width = 2000 bp, previous parameters were hotspot width = 300 bp, binding width = 700 bp). While the change in parameters was designed mainly to accommodate the wide and non-central binding pattern of RAD51, the same parameters were used for all three proteins to facilitate comparison. The correlation between previously published DMC1 measures and the estimates with these parameters for autosomal hotspots is 0.98. Heatmaps were prepared using Deeptools (Ramírez et al., 2016).

For each hotspot in the hybrid mouse, we also inferred the fraction of reads that originated from the B6 and the CAST chromosomes respectively, as previously described (Davies et al., 2016). For H3K4me3, previously published data on fractions of reads from each chromosome were used (Hinch et al., 2019). Asymmetric hotspots were defined as hotspots wherein the fraction of H3K4me3 reads originating on the B6 chromosome, denoted *f*, was either *f* ≥ 0.9 or *f* ≤ 0.1. Please also see legend for Figure S2F.

#### Deconvolution

Our raw data measures binding profiles of RAD51, DMC1, and RPA relative to the hotspot center (Figures 2A and S2C–S2E). Since DSBs occur over a few hundred base pairs around the hotspot center, the binding distributions are averages over the range of possible break sites. To get a more detailed mechanistic understanding of the underlying processes we wish to infer where these proteins bound relative to DSB sites. To this end, we perform deconvolution, a statistical procedure that takes into account the distribution of DSBs relative to the hotspot center, and is similar to the approach used in Lange et al. (2016) but with some differences.

Consider a set of hotspots H that have a unique well-defined PRDM9 motif with centers {*h*_*1*_*, h*_*2*_*, . . ., h*_*n*_}. Let $\bar{s_{i}}$ be the number of SPO11-oligos on either side of each hotspot center *h*_*i*_ in 20-bp bins: ${\bar{s}}_{i}$ = (*s*_*i,−m*_*, s*_*i,−(m−1)*_*, . . ., s*_*i,m−1*_*, s*_*i,m*_). Specifically, *s*_*i,l*_ is the number of SPO11-oligos between 20(*l* – ½) and 20(*l* + ½) bp from the hotspot center, and serves as an empirical estimate of the DSB frequency 20*l* bp from the hotspot center. The number of SPO11-oligos across all hotspots in the *l* th bin, denoted *s*_*∙,l*_ is therefore *s*_*∙,l*_ = ∑_*i∈{1,2,…,n}*_ *s*_*i,l*_ and across all bins is $\bar{s}\;$ = {*s*_*∙,−m*_*, s*_*∙,−(m−1)*_*, . . ., s*_*∙,m−1*_*, s*_*∙,m*_}.

Correspondingly, let $\bar{c}$ be the total binned coverage across all hotspots of one of the proteins in this study on the Crick strand, i.e., $\bar{c}$ = {*c*_*∙,−m*_*, c*_*∙,−(m−1)*_*, . . ., c*_*∙,m−1*_*, c*_*∙,m*_}, and $\bar{w}$ = {*w*_*∙,−m*_*, w*_*∙,−(m−1)*_*, . . ., w*_*∙,m−1*_ , *w*_*∙,m*_} on the Watson strand. We make a correction for background noise by subtracting the mean coverage distant from the hotspot center: for the Crick strand we estimate it as the mean coverage between 4000 bp and 5000 bp to the left of the hotspot center, and for the Watson strand we use the same distance to the right of the hotspot center. Any negative values are set to zero.

We wish to estimate $\bar{b}$, where *b*_*k*_ is the binding of this protein 20*k* bp from the DSB location in a manner sensitive to direction: we define positive values of *k* to have the same polarity as the 3′ ssDNA overhangs after resection, with negative values of *k* indicating the opposite direction. For example, in the event of perfect inference and no binding on the sister chromatid, we would expect *b*_*k*_ = 0 for *k* < 0 on the DSB-initiating chromosome. We restrict ourselves to estimating *b*_*k*_ between −1000 bp and 2500 bp from the DSB site (corresponding to *k* ∈ {−50,−49, . . ., 124, 125}), assuming it is zero elsewhere. The total protein binding at any particular distance from the hotspot center is the sum of the protein binding relative to the range of possible DSB sites. Therefore, for the Crick strand, ∀_*j*_ *c*_*∙,j*_ = ${\bar{t}}_{j}$ ∙ $\bar{b}$, where ${\bar{t}}_{j}$ is the transformed sequence of SPO11-oligo counts such that *t*_*j,k*_ *= s*_*∙,j−k*_*,* and correspondingly for the Watson strand. We confirmed empirically that the overall ChIP-seq signal is similar on both the Watson and Crick strands, with the ratios of the areas under the curves of the two strands being 1.001, 1.001, and 0.993 for RAD51, DMC1, and RPA respectively in the hybrid, and 0.996, 0.992, and 0.994 respectively in the wild-type.

This results in a system of overdetermined linear equations which we solve using least-squares minimization. Deconvolution is a standard problem in signal processing and can be implemented by a variety of methods (MacKay, 2003; Wasserman, 2007). We use ginv, an R procedure which calculates the Moore-Penrose generalized inverse of the matrix with the transformed SPO11-oligo counts, following Lange et al. (2016).To control over-fitting we set the tol parameter in ginv, defined as the relative tolerance to detect singular values, to 0.2.

SPO11-oligo maps are not available for the hybrid in this study, nor for any mouse with a PRDM9 allele different from PRDM9^B6^. Nevertheless, binding of different PRDM9 variants is known to result in a similar local chromatin landscape (Baker et al., 2014; Hinch et al., 2019; Lange et al., 2016) regardless of differences in sequence preferences and *a priori* chromatin conditions. We reasoned that, at least insofar as the deconvolution procedure is concerned, the average distribution of SPO11-oligo cuts for another PRDM9 allele may therefore be well-approximated by the distribution for the PRDM9^B6^ allele. To test this hypothesis, we used DMC1 data from the PWDxB6 mouse (Davies et al., 2016), which has the PRDM9^B6^ and PRDM9^PWD^ alleles, and performed deconvolution

for hotspots of both alleles separately, but using the average PRDM9^B6^ SPO11-oligo distribution in both cases. The inferred DMC1 binding relative to break sites was essentially identical for both PRDM9 alleles, thereby confirming that this approximation is appropriate. We leveraged the same average SPO11-binding distribution for hotspots in the hybrid mouse in this study as well.

Depending on the level of noise in the data and the tolerance for over-fitting set above, deconvolution can be unstable or imperfect. It seems likely that some of the (small) amount of signal on the wrong side of the break on the DSB-initiating chromosome after deconvolution (for example, in Figure 2B) is due to instability or uncertainty in deconvolution due to noise in data or due to the model above deviating from biological reality.

#### Structured Illumination Microscopy

Wild-type mice used in experiments were C57BL/6J (B6) background, or a mixed DBA2/B6 background. Juvenile mice (13-15 dpp) testes were collected for spermatocyte surface spreading and subsequent immunofluorescence. Two 5-month-old *Spo11*^−/−^ mice and two 3-month-old *Dmc1*^−/−^ mice were used in this study. The previously described *Spo11* mutation (Baudat et al., 2000) was maintained on a congenic B6 strain background. The *Dmc1* mutation (Pittman et al., 1998) was maintained on a mixed B6/ S129Sv/J background.

Primary antibodies: SYCP3 (1:200, mouse ab97672, Abcam), DMC1 (1:50, rabbit, sc-22768, Santa Cruz), DMC1 (1:50, guinea pig, home-made T113-1, ABclonal Biotechnology) and RAD51 (1:50, rabbit sc-8349, Santa Cruz). To raise polyclonal antibodies against DMC1, a fragment of mouse DMC1 coding sequence (corresponding to aa 1–100) was cloned into pET28a. This expression vector was used to produce purified 8 × His-DMC1 1–100 fragment in bacteria and then to immunize three guinea pigs by ABclonal Biotechnology Co., Ltd. Specificity of the DMC1 antibody was confirmed by immunoprecipitation from testis extracts of a protein of the appropriate molecular weight recognized in western blotting by an independent commercial anti-DMC1 antibody, and by absence of staining of chromosome spreads from a *Dmc1*^*–/–*^ mouse.

Secondary antibodies: goat anti-mouse IgG (H+L) Alexa Fluor 647 (1:200, Catalog# A-21235, Invitrogen); donkey anti-rabbit IgG (H+L), Alexa Fluor 555 (1:200, Catalog# A-31572, Invitrogen); donkey anti-rabbit IgG (H+L), Alexa Fluor 488 (1:100, Catalog# A-21206, Invitrogen); donkey anti-mouse IgG (H+L), Alexa Fluor 488 (1:100, Catalog# A-21202, Invitrogen); goat anti-mouse CF405S (1:200, Catalog# BT20080, Biotium); fluorescein (FITC)-conjugated affinipure donkey anti-guinea pig IgG(H+L) (1:100, 706-095-148, Jackson ImmunoResearch); goat anti-guinea pig IgG (H+L), Alexa Fluor 555 (1:200, Catalog# A-21435, Invitrogen); goat anti-guinea pig 594 (1:300, Catalog# A-11076, Invitrogen) and goat anti-guinea pig 488 (1:100, Catalog# A-11073, Invitrogen).

Spermatocyte chromosome preparations and immunofluorescence were performed as described previously (Jiang et al., 2017; Peters et al., 1997) with the following modifications. Slides were either used for immunofluorescence staining immediately or stored at −80°C. For immunofluorescence, slides were blocked for 35 min with 1x phosphate-buffered saline with 0.1% Triton X-100 (PBST) containing 3% nonfat milk. Slides were then incubated with indicated primary antibodies overnight at 4°C in a humidified chamber. Slides were then washed four to five times in 1x PBS with 0.1% Triton X-100 for 6 min each time. Secondary antibodies were applied for 1 hr at 37°C in a humidified chamber. Both primary and secondary antibodies were diluted in PBST containing 3% nonfat milk. After secondary antibody incubation, four to five washes were performed in PBST and the slides were mounted with VECTASHIELD mounting medium (H-1000, Vector Laboratories).

For data presented in Figures 3 and S3A–S3F, images were were captured using a high-resolution structured illumination microscopy (SIM) microscope (Nikon) equipped with objective lens (SR Apo TIRF 100x, NA 1.49) and connected to a CCD camera (Andor DU-897 X-11459), then images were processed using the NIS-Elements software (Nikon). Data presented in these figures were pooled from three independent staining experiments. In two experiments, secondary antibodies were labeled with FITC for DMC1 and Alexa-555 for RAD51. In the other experiment, the fluorophore colors were reversed (Alexa-555 for DMC1 and Alexa 488 for RAD51).

SIM images were analyzed with semi-automated scripts in Fiji (Schindelin et al., 2012). Briefly, the SYCP3 channel (Alexa-647) was first thresholded, segmented, and converted to a distance map. Then, the RAD51 and DMC1 channels were thresholded and the maxima were determined. These maxima were overlaid onto the distance map in order to determine their distance to the closest SYCP3 signal. All images were also manually inspected during the analysis to ensure that automated assignments were correct.

For the data in Figures S4 and S5, numbers of mice analyzed are indicated in the figure legend. For results of DMC1 specificity test (Figures S4A and S4B), conventional microscope images of spread spermatocytes were acquired on a Zeiss Axio Observer Z1 Marianas Workstation, equipped with an ORCA-Flash 4.0 camera and DAPI, CFP, FITC, Texas Red and Cy5 filter sets, illuminated by an X-Cite 120 PC-Q light source, with 100X/1.4 NA oil immersion objective. Marianas Slidebook (Intelligent Imaging Innovations) software was used for acquisition.

For experiments evaluating dye swap (Figure S5A) or testing colocalization with different DMC1 antibodies (Figure S5B), SIM was performed at the Bio-Imaging Resource Center in Rockefeller University using an OMX Blaze 3D-SIM super-resolution microscope (Applied Precision), equipped with 405 nm, 488 nm and 568 nm lasers, and 100X/1.40 NA UPLSAPO oil objective (Olympus). Image stacks of several μm thickness were taken with 0.125 μm optical section spacing, and were reconstructed in Deltavision softWoRx 7.0.0 software with a Wiener filter of 0.002 using wavelength specific experimentally determined OTF functions. Maximum intensity projection images were acquired in Deltavision softWoRx 7.0.0 software. Slides were prepared and stained as described above, except that chromosomes were spread only on the central portion of the slides, and the slides mounted using 20x20 mm coverslips.

To ensure correct alignment of fluorescent channels in all SIM experiments, multi-color fluorescent beads (TetraSpeck microspheres, 100 nm, Life Technologies, T7279) were imaged and used to correct shifts from passage of different fluorescence wavelengths through the optical path.

We observed that essentially all of the DMC1 foci are close to axes (97%, axes identified by SYCP3 staining), even when there is no detected RAD51 focus nearby. In contrast, many RAD51 foci are far from axes (19%). While the majority of axis-associated RAD51 foci have a DMC1 co-focus (82%), RAD51 foci away from the axes rarely have a DMC1 focus nearby (4%). Therefore, we hypothesized that these off-axis RAD51 foci are likely to be background (not programmed DSBs). This interpretation is supported by the observation that the distribution of distances of RAD51 foci from their nearest axis point, for those foci which are greater than about 450 nm from the nearest axis, is flat and resembles a uniform distribution. The uniform distribution is expected for the distances of points that are randomly scattered (i.e., noise) in a two-dimensional plane to the nearest point on a fixed line. To examine this further, we performed conventional immunofluorescence microscopy of spread spermatocytes stained with antibodies for RAD51 and SYCP3 in a *Spo11*^−/−^ mutant as described above. Note the presence of weak off-axis RAD51 foci (Figure S4B). This supports the interpretation that background staining with this antibody is higher; these foci may be non-specific cross-reaction of the antibody, or may reflect SPO11-independent complexes of RAD51.

We defined RAD51 and DMC1 foci to be within a co-focus if they are within 300 nm of a focus of the other protein. We chose this threshold based on the neighbor distances in Figure S3D. Other thresholds, between **∼**250 nm and **∼**350 nm also seem suitable. The distributions in Figure S3E are essentially unchanged by using either of these thresholds instead of 300 nm, and the results, i.e., that median RAD51 focus to axis distance = 60 nm, and median DMC1 focus to axis distance = 120 nm also remain the same.

#### Modeling RPA as a combination of DMC1 & RAD51

As discussed in the main text, the total ChIP-seq signal measured for the occupancy of a protein may be affected by experimental factors such as the affinity of an antibody for its target, the efficiency of cross-linking and immunoprecipitation, etc. Therefore, we cannot directly compare the numerical values of signals across experiments. Nevertheless, the shapes of the signals are expected to be robust to these differences. This was indeed found to be the case when the shapes of DMC1 distributions were compared for replicate experiments, as well as hotspots from the same PRDM9 allele in mice with different genotypes but otherwise similar passage through meiosis, e.g., PRDM9^HUM^ and PRDM9^PWD^ hotspots in PWD^HUM^xB6^HUM^ and B6^HUM^xPWD^PWD^ mice (the superscript refer to the PRDM9 variant activating the hotspots), and PRDM9^HUM^ hotspots in B6^HUM^xB6^HUM^ and B6^B6^xB6^HUM^ mice (Davies et al., 2016).

While the absolute values of the binding measures cannot be compared between experiments, we hypothesized that the shape of the distributions may be informative, and hence that the shape of the RPA binding density may reveal the properties of how it is displaced by DMC1 and RAD51. We use a simple linear model with intercept:

P=α+βD+γR

where *P, D* and *R* are the deconvolved distributions of RPA, DMC1 and RAD51 binding on the DSB-initiating chromosome in the hybrid. All three deconvolved distributions were normalized to have area 1 prior to the fitting. The linear model was fitted using the lm function in R for PRDM9^CAST^ and PRDM9^HUM^ hotspots separately. For PRDM9^CAST^ hotspots, the inferred values were α = 0.00041, β = 0.600, γ = 0.327, corresponding to relative DMC1 and RAD51 proportions of 0.65 (i.e., 0.600/(0.600 + 0.327)) and 0.35 (i.e., 0.327/(0.600 + 0.327)) respectively. For PRDM9^HUM^ hotspots, the inferred values were α = 0.00021, β = 0.599, γ = 0.364, corresponding to relative DMC1 and RAD51 proportions of 0.62 and 0.38 respectively. The same intercept was used to illustrate the optimal and alternative fits in Figure S6A.

To interpret the combination of ChIP-seq signals in terms of protein binding, we need to make additional assumptions. We assume that (1) the lifespan of the binding of a protein is the same for different parts of the resected single-strand DNA on average, and (2) RPA binds the DSB-initiating chromosome prior to DMC1 and RAD51 loading but not after they are removed later in prophase I. We do not have to make further assumptions about how long RAD51 and DMC1 remain bound (e.g., whether or not they are bound for the same length of time). Formally, it is not required that RPA be completely replaced by DMC1 and RAD51 as long as any remaining RPA binds the ssDNA with the same probability distribution as the RPA binding prior to being replaced by DMC1 and RAD51. In any case, our data indicate that RPA does not remain present at a significant level during the time DMC1 and RAD51 are bound (see main text; Figures 4C and S6B–S6H). However, we cannot rule out that RPA returns to bind the DSB-initiating chromosome after the removal of DMC1 and RAD51 from ssDNA once strand exchange has taken place.

In the event that this second assumption is incorrect, it remains the case that DMC1 and RAD51 fit the RPA signal precisely and this may be interpreted as follows. If any later RPA binding on the DSB-initiating chromosome is at a much shorter timescale than RPA binding prior to DMC1 and RAD51 loading, it will not meaningfully impact the results. On the other hand, if the late RPA binding on the DSB-initiating chromosome is long-lived, the precise model fit with DMC1 and RAD51 signals implies that one of the following occurs: (1) only RAD51 is replaced by RPA and this happens *throughout* the extent of RAD51 binding, or (2) only DMC1 is replaced by RPA and this happens *throughout* the extent of DMC1 binding, or (3) DMC1 and RAD51 are both replaced by RPA, and this happens *throughout* the extent of their binding. Among these possibilities, (2) and (3) seem unlikely: once strand-exchange has taken place, a segment of DNA that was previously bound by DMC1 is paired with a repair-template strand, and that segment of DNA should no longer available to be bound by RPA.

#### Skewed hotspots

We observed that hotspots often have more DMC1 binding on one side (left or right) of the hotspot center than the other. These hotspots also tend to have more RPA and RAD51 binding on the same side that has more DMC1 binding. To understand this systematically, we centered hotspots on their respective PRDM9 binding motif and compared the DMC1 coverage going from left to right (up to 5 kb in each direction). We calculated the point at which the coverage was 50% of the total for each hotspot (say *m*). We defined a hotspot to be skewed if *m* was > 150 bp from the hotspot center. A hotspot where *m* was to the left of the hotspot center was skewed to the left and conversely. We calculated the RPA and RAD51 coverage (for both mice) and SPO11-oligo coverage (for B6) for the same set of hotspots.

To compare the general properties of skewed hotspots, we flipped around hotspots that were skewed to the right, so that all skewed hotspots had more DMC1 to the left. The same set of hotspots was used consistently for all measurements: DMC1, RPA, RAD51, SPO11 oligos, non-crossovers, crossovers, and single-nucleotide polymorphisms.

In the construction of Figures S7C–S7E, we used published data for non-crossover tracts from Li et al. (2019). Data from all generations were pooled to maximize power. A non-crossover tract is defined as the minimal contiguous segment that was observed to have been converted. For example, for non-crossovers where only one SNP was converted, the tract was defined to be the location of that SNP. For multi-SNP non-crossovers, the full region between the two furthest converted SNP is defined to be the tract. The total weight given to all non-crossovers is the same, regardless of length or resolution.

We performed the following detailed checks and analyses to understand the underlying causes of the observed imbalance in the ChIP-seq signal:

• The imbalance in DMC1, RPA and RAD51 in skewed hotspots cannot be explained by imbalance in DSBs. In Figure 4D, the plots show the binding patterns relative to DSB sites (i.e., after deconvolution). Deconvolution was performed using the SPO11-oligo distribution obtained from the same set of (skewed) hotspots. These plots therefore fully take DSB localization into account and are not confounded by differences in DSB localization. To analyze this further, we restricted to hotspots with narrow and central DSB localization, specifically where fewer than a quarter of the DSBs occur more than 100 bp away from the center of the hotspot in either direction (Figure S7B). As seen in the bottom plots of Figure S7B, this successfully restricts to hotspots without significant imbalance in DSB localization and yet imbalance in RPA/DMC1/RAD51 is still observed.
• The imbalance in non-crossovers in skewed hotspots cannot be explained by imbalance in DSBs or in SNP density. In Figures S7D and S7E, we compared the distributions of non-crossover and SPO11 oligos in both skewed and balanced hotspots. In Figure S9B, we compared the distribution of SNPs in both skewed and balanced hotspots with non-crossovers in skewed hotspots. Note that the non-crossover distribution is more strongly skewed than the distribution of SPO11 oligos or SNPs in skewed hotspots.
• The findings cannot be explained by artifacts in mapping of sequencing reads. (i) Mapping artifacts due to the particular requirements of the single-stranded DNA sequencing (SSDS) assay: A sequencing read is identified as having come from an ssDNA fragment by inferring DNA hairpins due to microhomology from an inverted terminal repeat sequence (Khil et al., 2012). If the imbalance or the particular shapes of the DMC1 and RPA distributions on either side were due to differences in the presence of this sequence feature, we would expect to observe the same bias in the same set of hotspots regardless of how well or how quickly different strains of mouse are able to complete homologous pairing during prophase I (Figure S8B). However, we observe consistent and reproducible differences between mice, with the biggest skew between the two sides of a break observed in fertile mice and the smallest skew observed in completely infertile mice (Figure S8B). The link between the shape of the RPA and DMC1 distributions with the time taken to find the homolog (and the success rate in doing so) is also consistent with observations in different sets of hotspots between the B6 mouse and the hybrid mouse in this work: both DMC1 and RPA show a sharper increase near DSB sites in the B6 wild-type mouse than they do in the hybrid. (ii) Differences in mappability in general: The point above, in which the same set of hotspots are compared in different strains of mice, also proves that general differences in mappability cannot explain the observed differences in RPA, DMC1, and RAD51 signals. Second, the detection of non-crossovers is highly robust to differences in mappability of sequencing reads (see Li et al., 2019). Third, only subtle differences are observed in assays which measure activities that take place upstream of breaks (H3K4me3) or those measuring breaks (SPO11 oligos) (Figures S7B and S9C), while large differences are observed downstream of breaks. This is not predicted from a general difference in mappability of sequencing reads.
• The sharp increase in shapes of RPA and DMC1 very close to the DSB site (Figures 2B and 2D) is unlikely to be a sonication artifact. The idea behind the sonication bias hypothesis is that the DSB end of single-stranded DNA is already broken prior to sonication. Therefore, only one additional break from sonication is required for fragmenting the ssDNA to a smaller size. We note several arguments against this idea: first, the sharp increase in DMC1 and RPA appears to occur only on the less-bound side in skewed hotspots. If sonication was the underlying cause of this observation, both sides of a hotspot should be affected by it in the same way. Second, simulations under only very specific conditions reproduce the observed pattern (a single very high intensity pulse, data not shown). Yet, the results for DMC1 for genetically identical mice have been reproducible in different laboratories under a variety of sonication conditions, with different intensities and rounds of sonication, and using different sonication equipment. Third, both DMC1 and RPA show a greater increase near the DSB site in the B6 wild-type mouse than they do in the hybrid. This consistent difference between mice suggests an underlying feature that affects both DMC1 and RPA binding similarly. Thus our data indicate that the increase is not an artifact of sonication, and that it represents a consistent feature of recombination, which varies between genetically distinct mice in a reproducible manner.

One explanation for the skew in both binding and downstream repair outcomes in skewed hotspots is that proteins necessary for recombination are less able to load on the less-bound side. This may be due a difference in the efficiency of break processing and loading of RPA on the two sides, leading to differences in the subsequent loading of DMC1 and RAD51. A specific possibility is that there may be a delay after the initial resection of the nicked DNA in the 3′ to 5′ direction but before the action of the 5′ to 3′ exonuclease on the less-bound side (thereby leading to a short stretch of ssDNA near the break site). Since strand invasion is necessary for downstream repair outcomes, reduced loading of proteins required for strand invasion is expected to lead to fewer non-crossovers and crossovers on the less-bound side.

#### Time courses of DMC1 and RPA foci

For the comparison of the time courses of DMC1 and RPA foci, spermatocytes from adult mice were prepared for immunohistochemistry by surface spreading as previously reported (Davies et al., 2016). The following antibodies were used: rabbit anti-DMC1 (Santacruz Biotechnology sc-22768, H-100); biotinylated rabbit anti-SYCP3 (Novus, NB300-232); mouse anti-SYCP3 (Santacruz Biotechnology sc-74569, D-1); rabbit anti-RPA2 (Abcam ab10359). For the detection of DMC1 and SYCP3, the primary antibody anti-DMC1 was diluted 1:100 in B/ABD buffer (PBS 0.2% BSA, 0.2% gelatin, 0.05% Tween-20), applied onto the cells, and incubated for 30 minutes at 37°C. Following 4 washes of 5 minutes each in B/ABD buffer, AlexaFluor goat anti-rabbit 594 (ThermoFisher Scientific) and AlexaFluor donkey anti-mouse 488 (ThermoFisher Scientific) were diluted in B/ABD buffer 1:500, applied on the cells, and incubated 30 minutes at 37°C. Following 4 washes of 5 minutes each in B/ABD buffer, the biotinylated rabbit anti-SYCP3, was diluted 1:100 in B/ABD buffer, applied on the cells, and incubated 30 minutes at 37°C. After 4 washes of 5 minutes each in B/ABD buffer, and one wash in 4xSSC, 0.1% Tween-20, avidin Cy5 (ThermoFisher Scientific), diluted 1:50 4xSSC, 0.1% Tween-20, 3% non-fat dry milk, was applied to the cells, and incubated 30 minutes at 37°C. After four washes in 4xSSC, 0.1% Tween-20, and one wash in 0.1xSSC, the slides were mounted in DAPI/Vectashield (Oncor). For the detection of RPA and SYCP3, the primary antibody was diluted 1:500 and 1:100 respectively, and detected with AlexaFluor goat anti-rabbit 594 and AlexaFluor donkey anti-mouse 488.

Image acquisition was performed on a BX-51 upright wide-field microscope, equipped with a JAI CVM4 B&W fluorescence CCD camera, operated via the Leica Cytovision Genus software, as well as with a Leica DM6B microscope for epifluorescence, with a DFC 9000Gt B&W fluorescence CCD camera, operated via Leica LASX software.

Image analysis was performed using Fiji (ImageJ-win64) (Schindelin et al., 2012). For cell scoring, the following criteria were used to classify the prophase stage: (i) Leptotene: Short, discontinuous SYCP3 signals. (ii) Early Zygotene: Long, discontinuous SYCP3 signals, more than 1 but fewer than 10 autosome pairs synapsing. (iii) Mid Zygotene: Between 10 and 16 autosome pairs synapsed. (iv) Late Zygotene: At least 17 but fewer than 19 autosome pairs synapsing, no clear XY body. (v) Early Pachytene: 19 synapsed autosome pairs, clear XY body, thicker lateral element complex (as visualized by SYCP3 staining). (vi) Mid Pachytene: 19 synapsed autosome pairs, clear XY body, elongated SYCP3 signal (as visualized by SYCP3 staining). (vii) Late Pachytene: 19 synapsed autosome pairs, clear XY body, elongated SYCP3 signal, ending in knob-like structures (as visualized by SYCP3 staining). (viii) Diplotene: At least one autosome pair starting to de-synapse.

#### Crossovers and RPA on the repair template

We wished to ask whether the RPA-bound intermediates we detect represent sites that will be repaired as crossover, or as non-crossover, or could they go down either pathway? Several studies have examined the time course of RPA foci by immunohistochemical detection of the protein across meiotic stages, relative to other markers of DSBs such as DMC1 or RAD51 foci (see main text). They have inferred that most if not all autosomal DSBs form late RPA foci when the autosomes are synapsing and new DSBs are not expected to be formed. Our own immunocytological comparison of the time courses of DMC1 and RPA foci in the wild-type and hybrid mice agree with these findings (Figures S10B and S10C). It follows that these large numbers of RPA foci (> 100 in early pachytene) cannot all be resolved as crossovers (∼24 per male meiosis), and many if not most are resolved as non-crossovers.

To investigate whether the RPA-bound intermediates are destined to be resolved only as non-crossovers instead, we compared the proportion of crossovers and the signal for RPA on the repair template in hotspots with the same expected number of breaks but in different parts of the genome (Figures 5C, S10D, and S10E). Since the number of DSBs is not known in the hybrid (SPO11-oligo data are available only for B6 and certain mutants), we approximated this quantity. In Figure 5C, we used RPA on the DSB-initiating chromosome as a proxy for the number of DSBs in hotspots. Following (Hinch et al., 2019), we also used H3K4me3 on the DSB-initiating chromosome as an estimator of the number of breaks and the results are shown in Figure S10E.

We find that regions with greater crossover resolution also have higher repair-template RPA (see main text). If RPA foci were destined to be non-crossovers only, we would expect to see the opposite, i.e., lower repair-template RPA in hotspots with higher crossover resolution probability. That this is not the case implies that RPA foci cannot be specific to the non-crossover outcome.

On the other hand, they confirm the cytological findings that RPA on the repair-template chromosome is not exclusive to the crossover outcome. If RPA signal on the repair-template chromosome was entirely from crossover-destined intermediates, we would expect it to show a difference comparable to that in crossover outcomes between centromere-proximal and telomere-proximal hotspots. In contrast, the difference is subtle and significantly smaller than the difference between crossover outcomes (20% versus 280%, p < 10^−5^).

In these plots (Figures 5C, S10D, and S10E) we also indicate an estimated absolute probability of a DSB being resolved as a crossover. Whereas the number of crossovers per meiosis is now well estimated in male mouse, there is considerable uncertainty in the number of DSBs in individual cells (Martinez-Perez and Colaiácovo, 2009). Following Cole et al. (2012a) and Cole et al. (2012b) we used an estimate of 10% crossover resolution rate per inter-homolog intermediate on average. We then counted the total number of crossovers in each bin, and then re-scaled the results such that the average rate of crossover resolution in the hotspots examined was 10% across the bins.

To further understand the relationship between crossover resolution and the binding of RPA, DMC1, RAD51 and H3K4me3 on the DSB-initiating and repair-template chromosomes within hotspots, we performed linear modeling. We used a simple linear model with intercept:

$$C=\alpha +\eta_{d}H_{d}\;+\eta_{r}H_{r}+\beta_{d}P_{d}\;+\beta_{r}P_{r}\;+\gamma_{d}D_{d}\;+\gamma_{r}D_{r}\;+\rho_{d}R_{d}+\rho_{r}R_{r}$$

where *C, H, P, D* and *R* are the number of crossovers, and measures of H3K4me3, RPA, DMC1 and RAD51 respectively in a hotspot in the hybrid mouse. The subscript *d* refers to the DSB-initiating chromosome while *r* refers to the repair-template chromosome. We restricted to asymmetric PRDM9^CAST^ and PRDM9^HUM^ hotspots (approximately 20-fold difference between H3K4me3 levels on the two homologs on average) wherein the relative binding of all of these proteins to the two homologs could be determined (n = 2,024). Measurements of H3K4me3, RPA, DMC1 and RAD51 were all normalized to have mean 0 and variance 1. The data for crossovers was obtained from Hinch et al. (2019). The results of this linear modeling are reported in Table S3.

Due to their correlation with or dependence on the number of DSBs, these eight measures of recombination (H3K4me3, RPA, DMC1, and RAD51 on the DSB-initiating and repair-template chromosomes respectively) have overlapping information (formally, they are highly collinear). However, they also have independent information because these proteins participate at different stages in the process of recombination. Therefore, we wished to identify the minimal set of factors that are pertinent for predicting the number of crossovers in a hotspot. We performed variable selection using stepwise regression using the Aikake Information Criterion (AIC) (Hastie and Pregibon, 1992). We used the implementation step, which is an R procedure (see also Table S4).

The results in the main text and in Tables S3 and S4 show that repair-template RPA is higher when the crossover outcome is more likely. In order to quantify the relative lifespans of crossover and non-crossover destined intermediates, we have to make further assumptions. To begin with, we assume that (1) among inter-homolog recombination intermediates, 10% are resolved with a crossover and the remainder with a non-crossover (2) all DSBs are repaired either as a crossover or as a non-crossover, i.e., no sister repair (we relax this assumption later, see below), and (3) the average lifespan of crossover-destined intermediates is the same in different chromosomal regions and the same is true for non-crossover destined intermediates. In this scenario, we can express RPA on the repair-template chromosome in each bin as a linear combination of the lifespans of crossover and non-crossover destined intermediates weighted by the probability of a break being resolved as a crossover or a non-crossover respectively, in each bin. We solve this system of equations using least-squares minimization.

The results of this analysis indicate that crossover-destined intermediates persist on average ∼2.6 times longer than non-crossover destined intermediates. We get comparable results (2.5-fold) when we use the more stringent set of asymmetric hotspots (shown in Figure S10D). The estimate is 3-fold if we use H3K4me3 as a proxy for the number of breaks (Figure S10E).

We estimated the difference in the lifespan of crossover and non-crossover destined intermediates in other plausible biological scenarios: (1) If the fraction of inter-homolog intermediates resolved with a crossover is 15%, the estimate is 2-fold higher crossover lifespan. (2) Permitting sister repair: we used H3K4me3 as a proxy for DSBs to avoid biasing the results in this case and assumed equal probability of sister repair in each of the four bins: (a) With 5% probability of sister repair and 10% of inter-homolog intermediates repaired with a crossover: 3-fold (b) With 10% probability of sister repair and 10% of inter-homolog intermediates repaired with a crossover: 2.9-fold (c) With 20% probability of sister repair and 10% of inter-homolog intermediates repaired with crossover: 2.8-fold (d) With 50% probability of sister repair and 10% of inter-homolog intermediates repaired with crossover: 2.5-fold.

#### Crossover and non-crossover gene-conversions

To estimate crossover gene-conversion tracts we utilized previously published crossover break-point locations identified from sequencing of individual sperm from a mouse that is genetically identical to the hybrid mouse in this study (Hinch et al., 2019). We restricted to crossovers that overlapped hotspots wherein a unique high-quality PRDM9 motif match was found. We further restricted to crossovers for which the break-point was resolved within 1000 bp. This yielded 1,021 crossovers with a median resolution of 425 bp.

Following Hinch et al. (2019), we constructed a map of the localization of crossover breakpoints by assigning, for each crossover, an equal probability of the break-point occurring in any inter-SNP interval within the two informative SNPs marking the change in haplotype. Next, we aggregated this map to construct an average profile of the crossover break-point probability relative to the PRDM9 motif center by adding the probability mass for each crossover at every distance (1 bp windows) up to 2000 bp from the motif center in each direction. To transform this map into one containing the average profile of crossover break-points relative to DSB sites, we performed deconvolution. We used essentially the same approach as for deconvolution of the protein binding distributions as described above, with the only difference being that we fit a single distribution (as opposed to two (Watson and Crick) distributions for protein binding).

This map reflects the probability that a point, which is at distance *d* from a DSB, is a crossover break-point. From this map, we wish to estimate a *different* map: for every point distance *d* from a DSB, we wish to estimate the probability that that point is *contained within a crossover gene-conversion tract*. In principle, this requires knowledge of both end-points for each crossover. In these data, and indeed in any crossover data inferred from sperm or pedigree sequencing, only one of the four gametes from each meiosis is obtained. This implies that only one of the two endpoints for each crossover is obtained. If we assume, however, that crossover gene-conversion tracts overlap the sites where DSBs occurred, we can perform this calculation by treating each crossover break-point independently and adding up the tracts from the DSB site to the crossover-breakpoint (after weighting according to the probability of each site being a crossover-breakpoint). The assumption that a crossover gene-conversion tract overlaps its DSB site seems to be a reasonable one given current models for double Holliday junction formation and resolution (Brown and Bishop, 2014; Hunter, 2015) and findings from 12 reciprocal crossovers for which both endpoints could be identified by tetrad analysis in mouse (Cole et al., 2014). Tetrad analysis revealed that the vast majority of crossover gene-conversion tracts overlapped the hotspot center itself (9 out of 12) and 2 further crossovers were approximately the distance to the secondary SPO11-oligo peaks (Lange et al., 2016). The number of crossovers (2 out of 12) near these secondary peaks is also consistent with the relative frequency with which DSBs originate in secondary peaks relative to the main central DSB peak on average (∼20%; Lange et al., 2016). The final crossover tract is also likely to have overlapped a DSB, as the particular hotspot in this analysis (the so-called *A3* hotspot) is very intense and has a wide spread of DSBs (Lange et al., 2016). Thus at least 11 out of 12 (92%), and potentially all of the crossover gene-conversion tracts, are consistent with having overlapped DSB sites.

We used published data for non-crossover tracts from Li et al. (2019). The non-crossovers were identified in F2 mice and occurred in male and female F1 mice, of which the male mice were genetically identical to the hybrid in this study. The female mice were genetically identical with the exception of one sex chromosome (CAST X versus CAST Y chromosome). We restricted to non-crossovers that were within 2 kb of hotspots wherein a unique high-quality PRDM9 motif match was found, providing 124 non-crossovers. Deconvolution was performed as above for crossovers after smoothing (100 bp moving window).

### Quantification and Statistical Analysis

Statistical analyses were performed using R versions 3.1 to 3.5 (http://www.r-project.org). Statistical parameters and tests are reported in the Figures and corresponding Figure Legends.

## Supplemental Information

Document S1. Figures S1–S10 and Tables S1–S4

Document S2. Article plus Supplemental Information
