# Supplementary material for: The Configuration of RPA, RAD51, and DMC1 Binding in Meiosis Reveals the Nature of Critical Recombination Intermediates
Source: Mol Cell. 2020 Aug 20;79(4):689–701.e10. doi: 10.1016/j.molcel.2020.06.015 (PMC7447979; doi:10.1016/j.molcel.2020.06.015)
Supplement: Document S1. Figures S1–S10 and Tables S1–S4 [file mmc1.pdf]

Molecular Cell, Volume *79*

Supplemental Information

The Configuration of RPA, RAD51, and DMC1 Binding in Meiosis Reveals the Nature of Critical Recombination Intermediates

Anjali Gupta Hinch, Philipp W. Becker, Tao Li, Daniela Moralli, Gang Zhang, Clare Bycroft, Catherine Green, Scott Keeney, Qinghua Shi, Benjamin Davies, and Peter Donnelly

Supplementary Figure S1

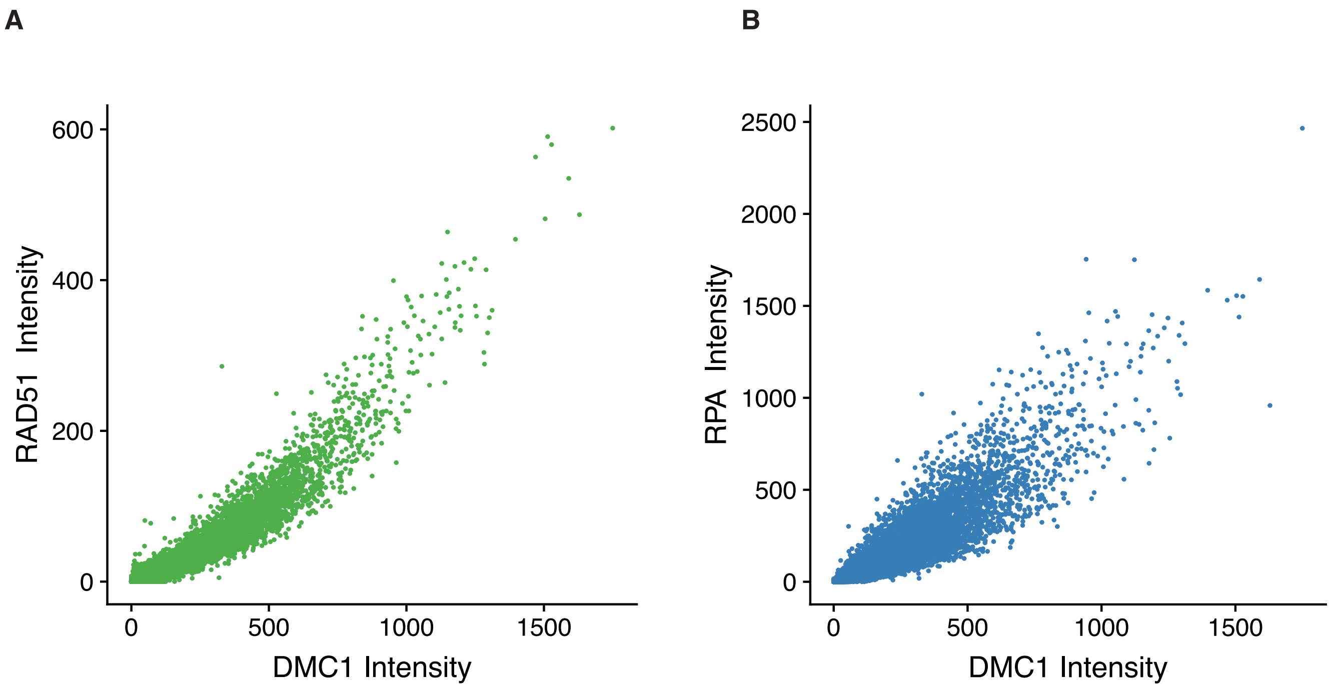

Figure S1 related to Figure 1. Comparison of RAD51, RPA and DMC1 measures in the B6 mouse
(A, B) Comparison of measures of RAD51 and RPA (A and B, respectively) with DMC1 binding (data from Brick et al., 2012) in autosomal recombination hotspots identified using DMC1 (n=16,926) in the B6 wild-type mouse.

Supplementary Figure S2

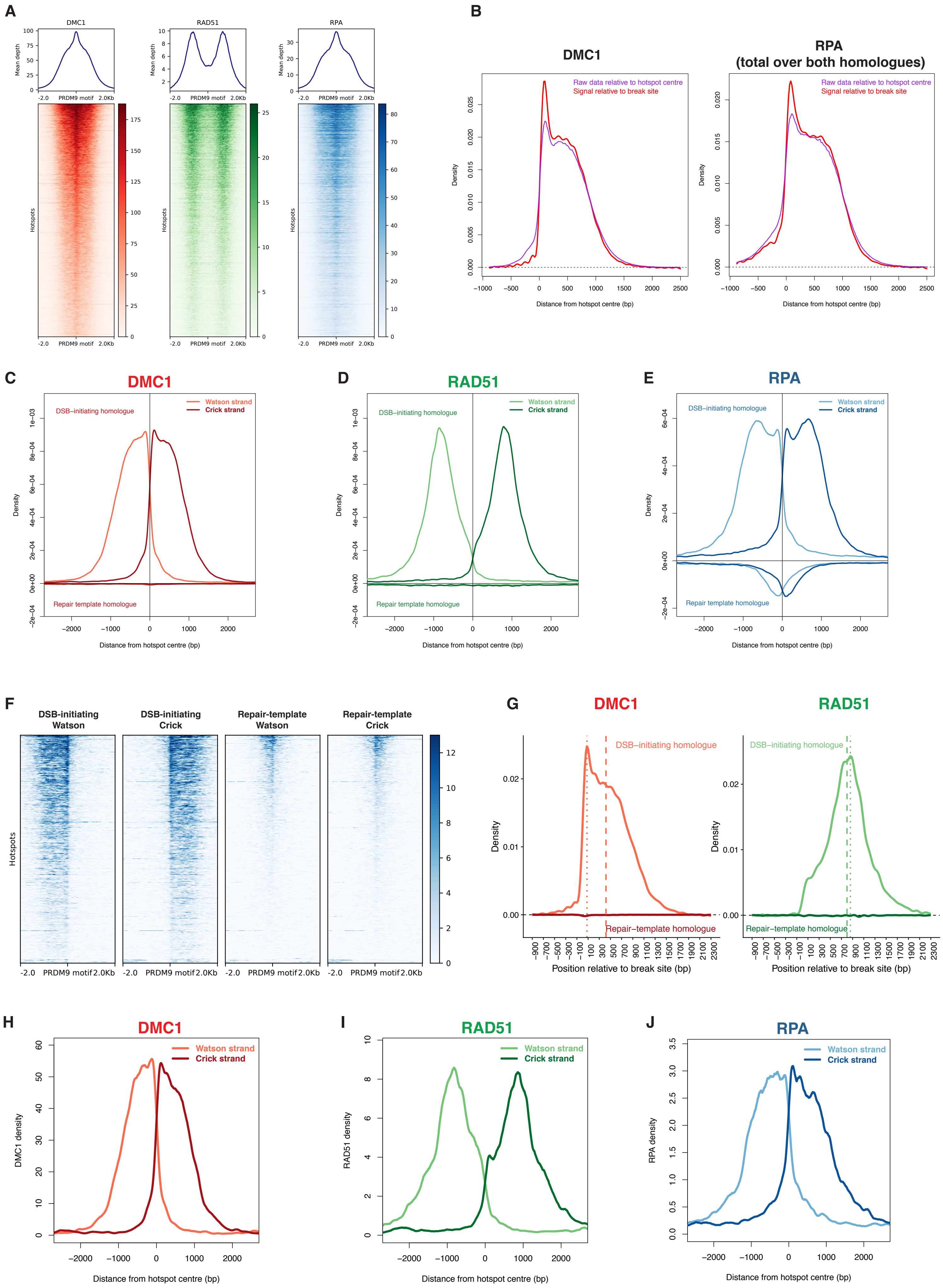

Figure S2 related to Figure 2: Binding of RPA, RAD51 and DMC1 in the hybrid mouse.

(A) (As in Fig. 2A, but for the hybrid mouse) Heat maps showing the read coverage of DMC1 (red), RAD51 (green), and RPA (blue) in recombination hotspots in the hybrid mouse that are activated by $PRDM9^{CAST}$. Each row is a hotspot and the 4000 most active hotspots with a well-defined PRDM9-binding motif are shown, ordered by the measurement of DMC1 in them (in descending order from top to bottom).
(B) Inferring protein binding relative to a break site. We infer the binding of proteins relative to a DSB (red), DMC1 (left) and RPA (right) (on the Crick strand in these illustrations) using the measured binding of proteins relative to the hotspot centre (purple) together with the known distribution of DSBs (Methods).
(C-E) *Raw data shown for completeness*:
(C) Raw data without deconvolution for the density of DMC1 binding relative to hotspot centres. The DSB-initiating (above the x-axis) and repair template (below the x-axis) homologues, separated for the Watson (light red) and Crick (dark red) strands in the hybrid mouse in asymmetric $PRDM9^{CAST}$ hotspots with a well-defined PRDM9 binding motif. The hotspot centre is defined as the centre of the PRDM9 motif site. Data are normalized so that the area under the curves for each strand is 1.
(D) As (C), but for RAD51 binding on the DSB-initiating (above the x-axis) and repair-template (below the x-axis) chromosomes, separated for the Watson (light green) and Crick (dark green) strands.
(E) As (C) but for RPA binding on the DSB-initiating (above the x-axis) and repair-template (below the x-axis) chromosomes, separated for the Watson (light blue) and Crick (dark blue) strands.
(F) Heat maps of RPA for the DSB-initiating and repair-template homologues in asymmetric $PRDM9^{CAST}$ hotspots where breaks are on average 20-fold more likely to originate on the B6 chromosome than on the CAST chromosome. Note that, unlike the density plots in this paper, these raw heat maps are not, by definition, corrected for the power to assign reads to homologues and should be interpreted with caution. We assign a read to a homologue by identifying whether its sequence matches CAST or B6 alleles at known polymorphic sites (Davies et al., 2016; Hinch et al., 2019). If a read maps to a region where no such polymorphism exists, it cannot be assigned to a homologue. Therefore, the power to assign reads depends on SNP density, which is often higher close to the PRDM9 motif in $PRDM9^{CAST}$ hotspots (Hinch et al., 2019; Li et al., 2019). This can increases the apparent signal close to the hotspot centre. It is essential to correct for biases due to differences in SNP density, and we do this by taking into account the total number of reads mapped relative to those that could be assigned to a homologue at every point in the genome. The density plots throughout this paper are thus corrected for differences in power at each location and at nucleotide resolution.
(G) As in Fig. 2B-C with DMC1 (left) and RAD51 (right) (Crick strand shown for both, 60 bp smoothing), together with the points at which the signals peak (dotted lines) and the medians of the distributions (dashed line). The median represents the point at which there is equal signal on either side.
(H,I,J) Density of DMC1, RAD51 and RPA binding respectively relative to hotspot centres (i.e., without deconvolution) in hotspots on the non-psuedoautosomal portion of the X chromosome.

Supplementary Figure S3

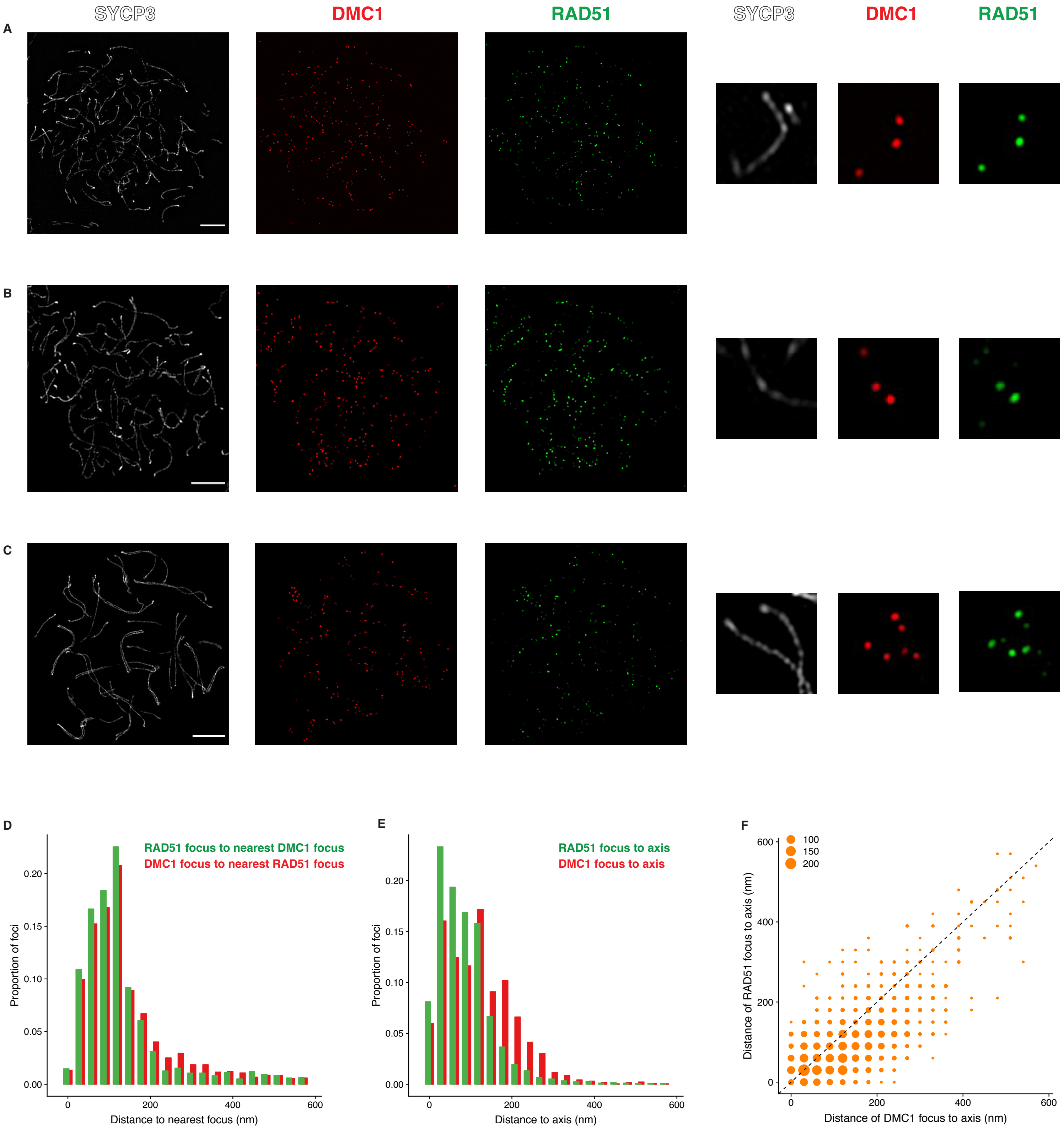

Figure S3 related to Figure 3: Structured illumination microscopy (SIM) of RAD51 and DMC1 foci.

(A-C) Separate immunofluorescence channels for SYCP3 (white), DMC1 (red) and RAD51 (green) staining corresponding to the images in Fig. **3(A-C),** respectively. Scale bars are 5 µm.
(D) Histogram of distances between each RAD51 focus to the nearest DMC1 focus (green), and of each DMC1 focus to the nearest RAD51 focus (red) from SIM microscopy of spread spermatocytes. For each axis-associated RAD51 and DMC1 focus (defined as being less than 450 nm of the nearest axis segment), the centroid-to-centroid distance was measured to the nearest focus of the other protein (3855 RAD51 and 4207 DMC1 foci analysed). Foci that were 450 nm or further from the nearest axis segment were analysed separately (Methods).
(E) Comparison of histograms of the distance to the nearest segment of SYCP3-defined axis for RAD51 (green) and DMC1 (red) foci from SIM microscopy of spread spermatocytes. Distances were compared for foci occurring within RAD51-DMC1 co-foci, i.e., those that have a focus of the other protein within 300 nm. RAD51 foci were significantly closer to the nearest axis than DMC1 foci ($p = 10^{-15}$), with the median distances being 60 nm for RAD51 and 120 nm for DMC1 foci.
(F) As in panel **E**, but directly comparing, within paired RAD51-DMC1 co-foci, the distance of DMC1 to the nearest axis segment (x-axis) with RAD51 to the nearest axis segment (y-axis). The size of each point is proportional to the number of co-foci at the corresponding distance from the axis. Amongst uniquely paired RAD51-DMC1 co-foci (n=3,139), RAD51 was closer to the axis in 1925 (61%), DMC1 was closer to the axis in 651 (21%) and both were equidistant (within the resolution of the assay) in 560 (18%). For visual reference, the dashed black line passes through the origin and has slope 1 (i.e., where RAD51 and DMC1 co-foci would lie if each protein were equidistant from the axis).

Supplementary Figure S4

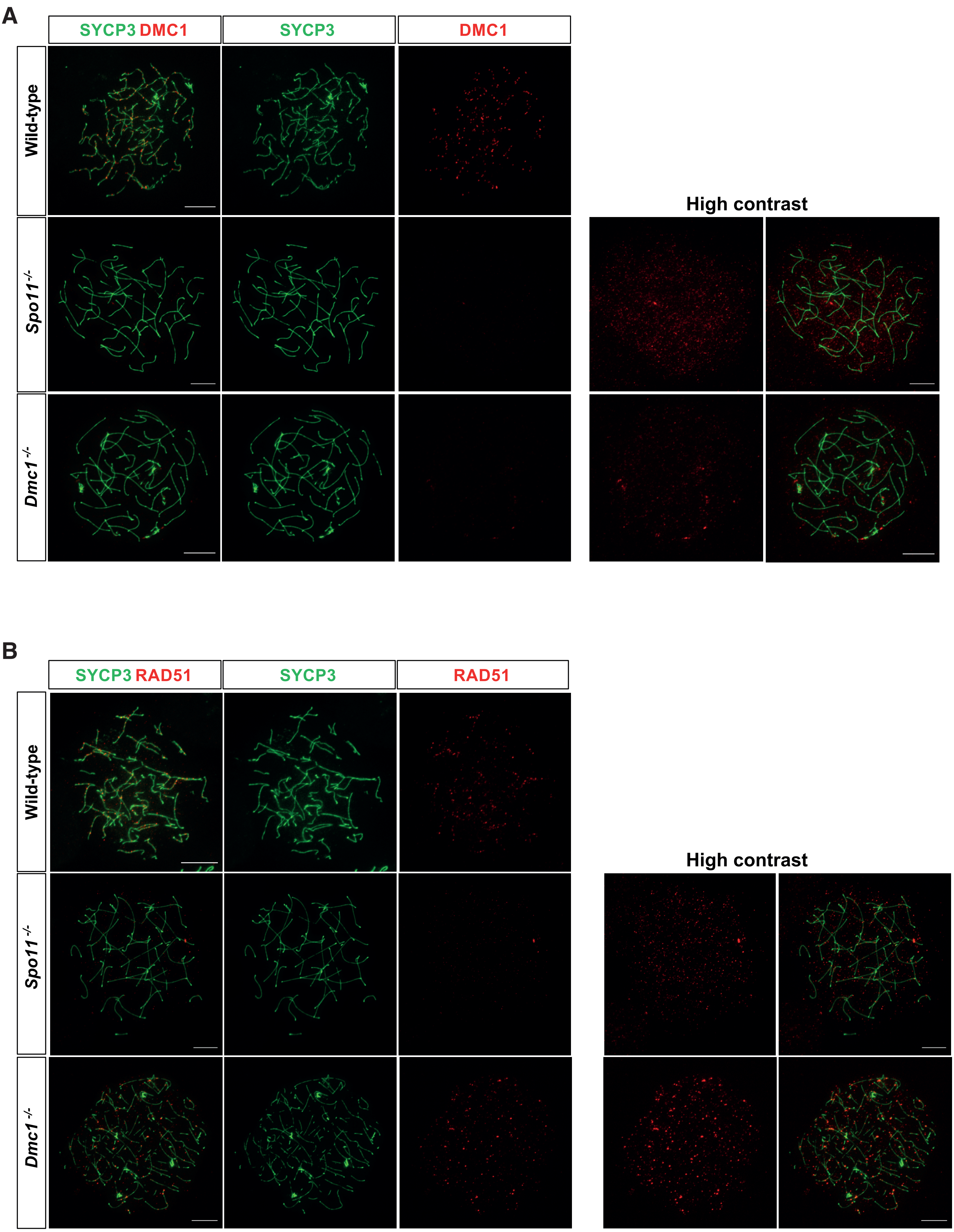

Figure S4 related to Figure 3: Confirming antibody-specificity for microscopy of RAD51 and DMC1 foci.

(A) Specificity of the home-made guinea pig anti-DMC1 antibody. Representative images from conventional immunofluorescence microscopy of spread spermatocytes stained with antibodies for DMC1 (red) and SYCP3 (green) in the wild-type (top row), and in *Spo11*$^{-/-}$ (middle row) and *Dmc1*$^{-/-}$ (bottom row) mutants. A brighter exposure of the DMC1 channel is shown to the right; note the higher background staining of chromatin in the *Spo11*$^{-/-}$ mutant than in *Dmc1*$^{-/-}$, as expected because the latter lacks DMC1 protein entirely. Mice were 3 to 5 months old. Wild-type and *Spo11*$^{-/-}$ were congenic with B6; the *Dmc1*$^{-/-}$ mutant is on a mixed (B6 x S129Sv/J) background. Representative images are shown from experiments performed on two mice of each genotype. Scale bars are 10 µm.

(B) As in panel **A**, but with a commercial rabbit anti-RAD51 antibody instead of anti-DMC1. Note the presence of weak off-axis RAD51 foci in the *Spo11*$^{-/-}$ mutant. These may be non-specific cross reaction of the antibody, or may reflect SPO11-independent complexes of RAD51.

Supplementary Figure S5

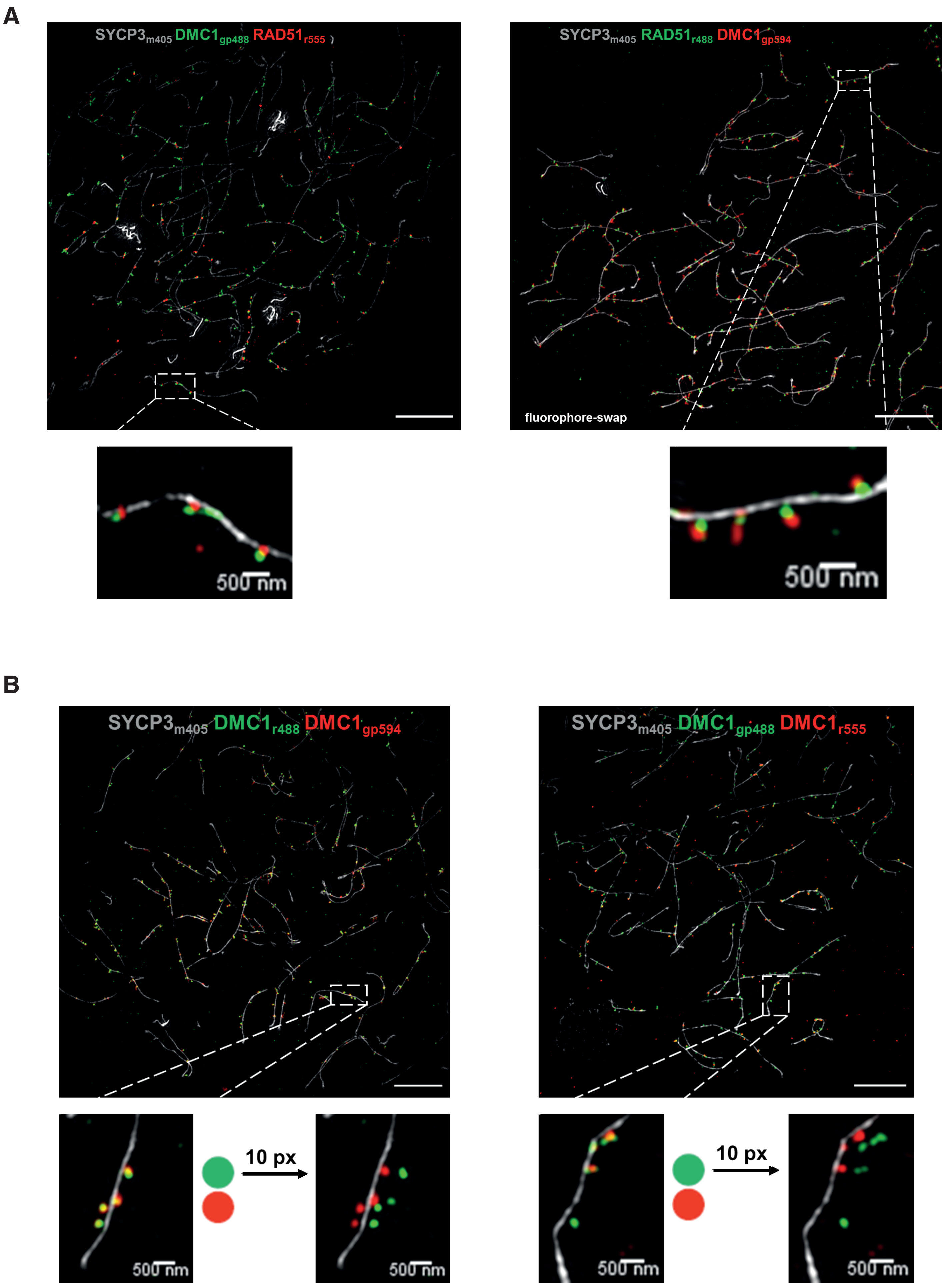

Figure S5 related to Figure 3: Fluorophore-swap experiment and comparison of staining with two distinct anti-DMC1 antibodies.

(A) Fluorophore-swap experiment. SIM images are shown of early zygotene spermatocytes from juvenile (13 dpp) B6 mice. To control for differences in fluorophore emission wavelengths, we switched secondary antibodies to detect guinea pig anti-DMC1 and rabbit anti-RAD51 primary antibodies. On the left, secondary antibodies were donkey anti-guinea pig Alexa 488 (green) and donkey anti-rabbit Alexa 555 (red). On the right, secondary antibodies were donkey anti-rabbit Alexa 488 (green) and goat anti-guinea pig Alexa 594 (red). Note that the anti-RAD51 staining is consistently more axis-proximal regardless of secondary antibody fluorophore (axes detected with mouse anti-SYCP3 primary and goat anti-mouse CF405S). Images are representative of experiments from two mice. Scale bars are 500 nm.
(B) Comparison of staining with two distinct anti-DMC1 antibodies. To further test specificity of the observed offset of anti-RAD51 and anti-DMC1 staining, we performed SIM on spermatocytes stained with two different anti-DMC1 antibodies: the home-made guinea pig antibody used throughout this study and a commercially available rabbit antibody. The panel on the left below each image shows a merged zoomed-in image with correctly aligned channels. The panel on the right shows the same section of the microscope field with the green channel shifted 10 pixels over. Images are representative of experiments from two 13-dpp B6 mice. Scale bars are 500 nm.

Supplementary Figure S6

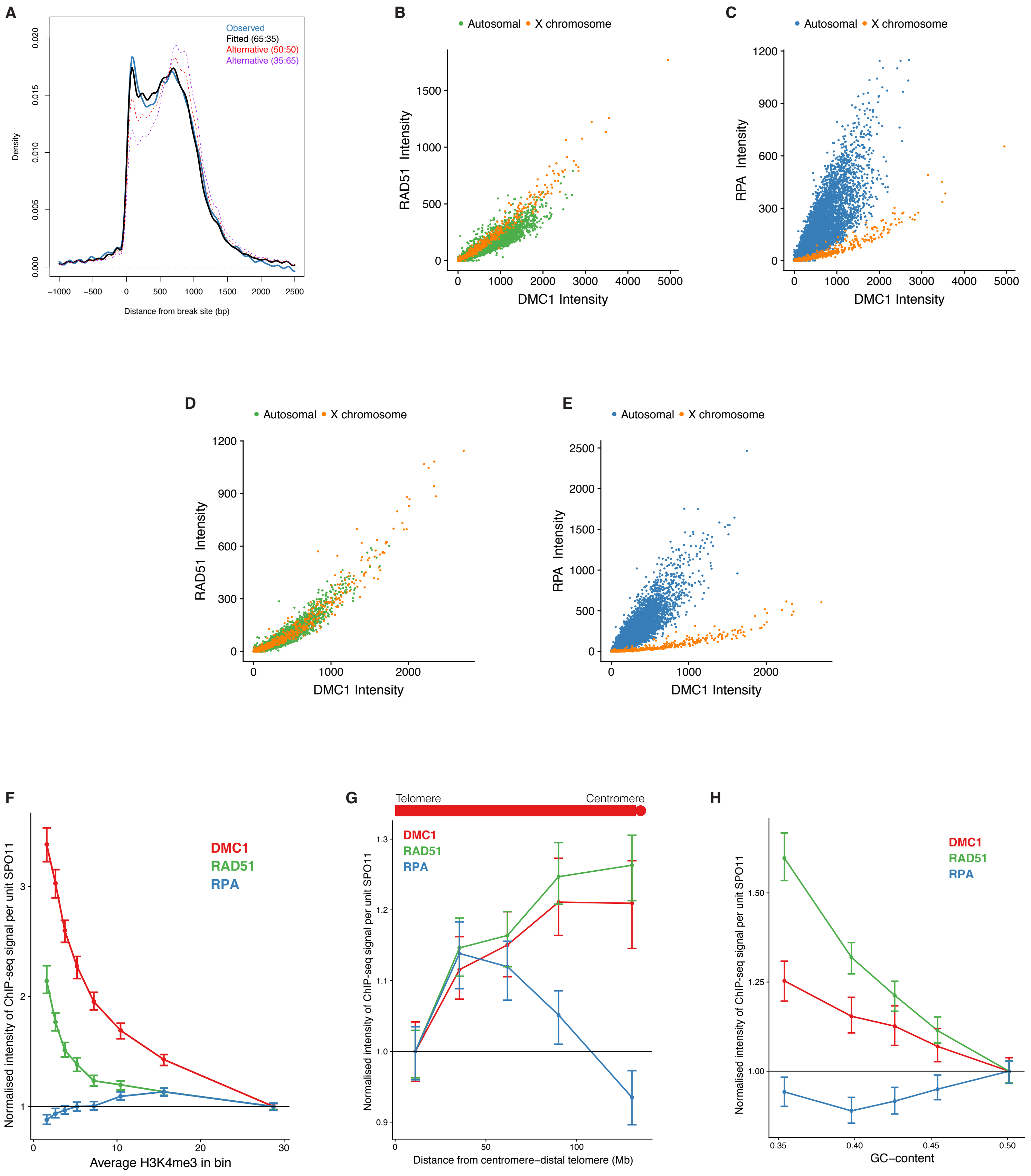

Figure S6 related to Figure 4: Relative binding of RPA, RAD51 and DMC1.

(A) Comparison of observed binding of RPA on the DSB-initiating chromosome with different combinations of DMC1 and RAD51 binding. Actual RPA binding (blue) compared with the best-fitting linear combination of DMC1 and RAD51 (black), together with alternative linear combinations, including one with equal proportions of DMC1 and RAD51 (dashed red) and the opposite proportions of DMC1 and RAD51 (35% and 65% respectively, dashed purple).
(B, C) Comparison of the measures of RAD51 and RPA binding (y-axis, B and C, respectively) with DMC1 binding (x-axis) in hotspots in the hybrid mouse, with X chromosome hotspots (n=906) in orange and autosomal hotspots (n=23,631) in green and blue, respectively.
(D, E) As in **(B, C)** but for the B6 wild-type mouse. Comparison of the measures of RAD51 and RPA binding (D and E, respectively) with DMC1 binding in hotspots in the B6 wild-type mouse, with X chromosome hotspots (n=1,103) in orange and autosomal hotspots (n=16,926) in green and blue, respectively.
(F) Whereas RAD51 and DMC1 are lower per break in hotspots that have high H3K4me3 and consequently engage with their homologue more quickly (Hinch et al., 2019), RPA does not show such a reduction. Autosomal PRDM9-dependent hotspots in the B6 mouse were ordered by their H3K4me3 intensity and divided into 8 bins. Average ratio of total DMC1 (red), RAD51 (green) and RPA (blue) to the total number of SPO11 oligos per bin, shown relative to the average H3K4me3 per hotspot in each bin; error bars denote 95% confidence intervals. Each protein measure was normalised such that the average ratio in the rightmost bin is 1. Although it would be ideal to restrict this analysis to the DSB-initiating homologue only, which is not possible here, our conclusion is supported by the large observed effect sizes and the observation that the majority of RPA signal arises from the DSB-initiating chromosome (~85%) in autosomal hotspots in the hybrid (Fig 2D).
(G) Whereas RAD51 and DMC1 are higher per break in hotspots that are further from the centromere-distal telomere and are slower to engage with their homologue (Hinch et al., 2019), RPA does not exhibit any consistent trend. Autosomal PRDM9-dependent hotspots in the B6 mouse were divided into five bins depending on their distance from the distal telomere of their respective chromosome. Average ratio of total DMC1 (red), RAD51 (green) and RPA (blue) to the total number of SPO11 oligos per bin is shown, error bars denote 95% confidence intervals. Each protein measure was normalised such that the average ratio in the leftmost bin is 1.
(H) Whereas RAD51 and DMC1 are lower per break in hotspots with high GC-content (Hinch et al., 2019), RPA does not show any consistent trend. Autosomal PRDM9-dependent hotspots in B6 were divided into five bins depending on their local GC content (within 500 bp from the hotspot centre). Average ratio of total DMC1 (red), RAD51 (green) and RPA (blue) to the total number of SPO11 oligos per bin shown; error bars denote 95% confidence intervals. Each protein measure was normalised such that the average ratio in the rightmost bin is 1.

Supplementary Figure S7

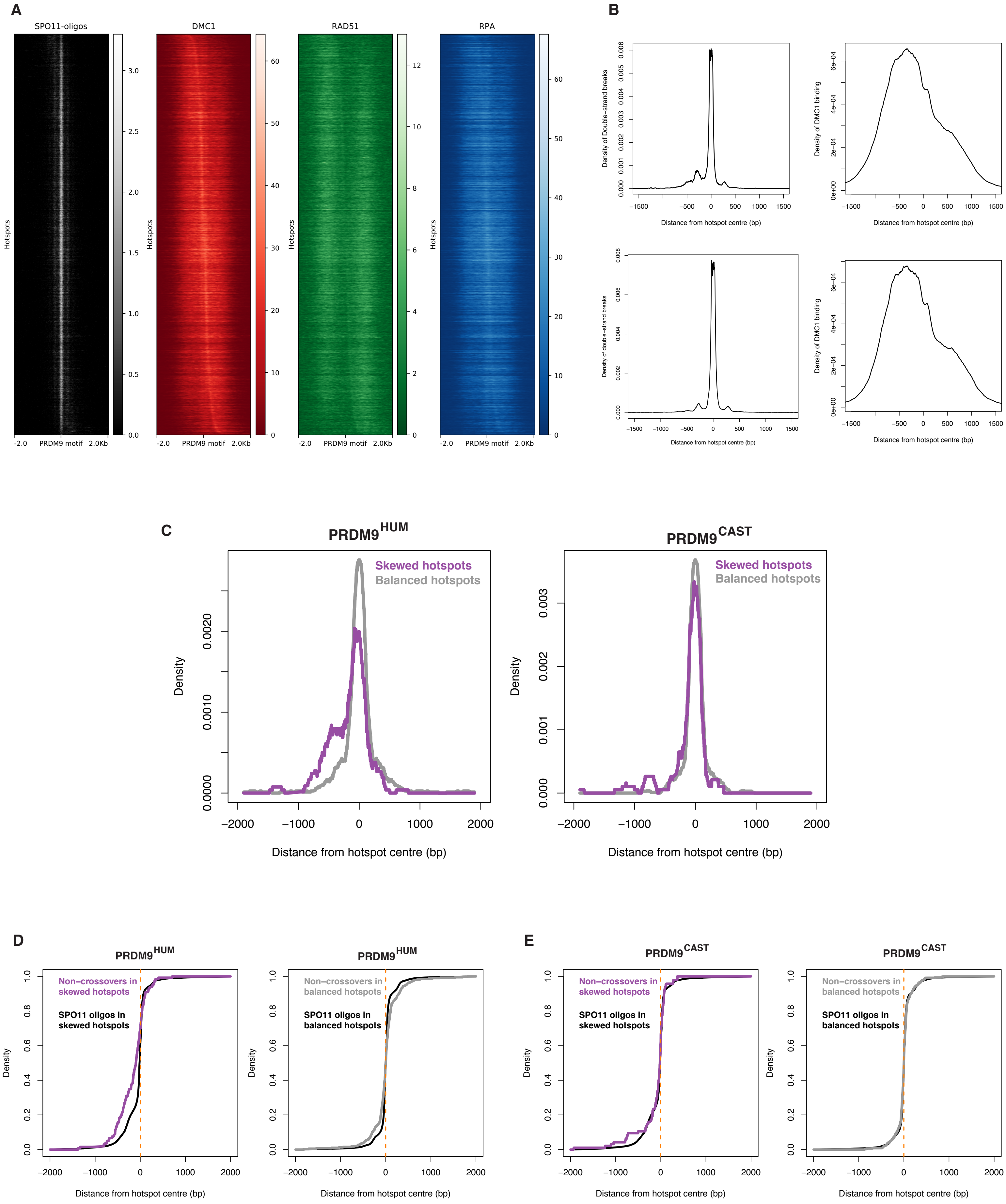

Figure S7 related to Figure 4: Heat maps of protein binding and localization of non-crossovers and double-strand breaks in skewed hotspots.

(A) Protein binding in skewed hotspots. Heat maps of SPO11 oligos, DMC1, RAD51 and RPA in autosomal B6 hotspots with a well-defined $PRDM9^{B6}$ motif that were more than 4 kb away from the nearest hotspot (hotspots defined using DMC1, n=15,893). Each row is a hotspot, and hotspots were ordered by the distance of the median point of the DMC1 ChIP-seq signal to the mid-point of the PRDM9 binding motif.
(B) Imbalance in DMC1 signal is not explained by imbalance in DSB localization in skewed hotspots. (Top row) Distribution of DSBs (measured by SPO11 oligos) (left) and DMC1 (right) in skewed DMC1 hotspots. Hotspots were co-oriented so that the side with more DMC1 was to the left of the hotspot centre. (Bottom row) To prevent confounding with DSB localization, only hotspots without significant skew in the location of DSBs were included. Distribution of DSBs (left) and DMC1 (right) in these hotspots. DMC1 hotspots continue to be strongly skewed after removal of hotspots with (slight) imbalance in DSBs.
(C) Localisation of non-crossover gene-conversion tracts in $PRDM9^{HUM}$ (left) and $PRDM9^{CAST}$ (right) hotspots (non-crossover data from Li et al., 2019). Skewed hotspots are shown in purple (132 and 94 non-crossovers in $PRDM9^{HUM}$ and $PRDM9^{CAST}$ hotspots respectively) and have a statistically significant shift in non-crossovers ($p = 5x10^{-7}$ jointly, $p = 7x10^{-6}$ for $PRDM9^{HUM}$ and p=0.017 for $PRDM9^{CAST}$). Balanced hotspots are shown in grey (258 and 301 non-crossovers in $PRDM9^{HUM}$ and $PRDM9^{CAST}$ hotspots respectively). Skewed hotspots were co-oriented such that the side with more DMC1 is to the left, balanced hotspots are shown averaged for both sides. Only non-crossovers within 2 kb of hotspots with a unique well-defined PRDM9 motif were included. Hotspots that are within 8 kb of another hotspot and complex non-crossovers were excluded. The area under the curve is normalised to be 1 for all curves.
(D) Comparison of the localization of non-crossovers in $PRDM9^{HUM}$ hotspots with SPO11 oligos in skewed (left, purple) and balanced (right, grey) hotspots. Skewed hotspots were co-oriented such that the side with more DMC1 is to the left. To assess whether the shift in non-crossovers can be explained by an imbalance in SPO11 oligos (e.g., due to a weak undetected hotspot nearby), we compared the cumulative distributions of non-crossover gene-conversion tracts (purple/grey) relative to SPO11 oligos (black) going from left to right within hotspots. Non-crossovers tracts are more strongly shifted to the more-bound side than DSBs, and therefore cannot be explained solely by a shift in DSB localisation in skewed hotspots.
(E) As in panel **D** but for non-crossovers in $PRDM9^{CAST}$ hotspots.

Supplementary Figure S8

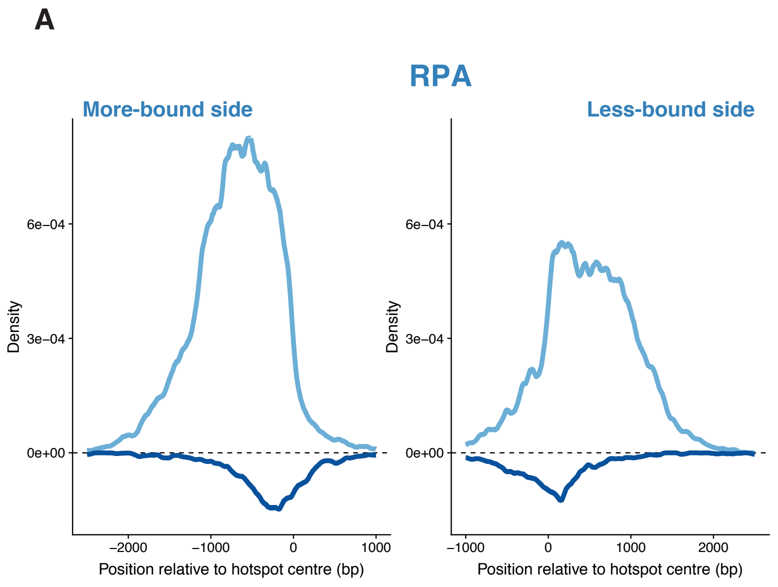

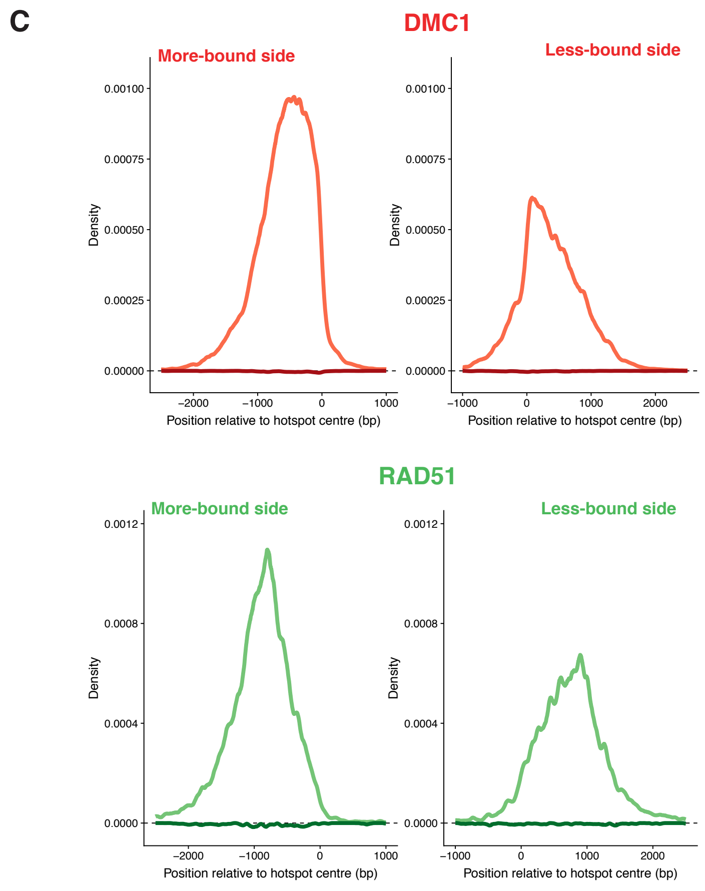

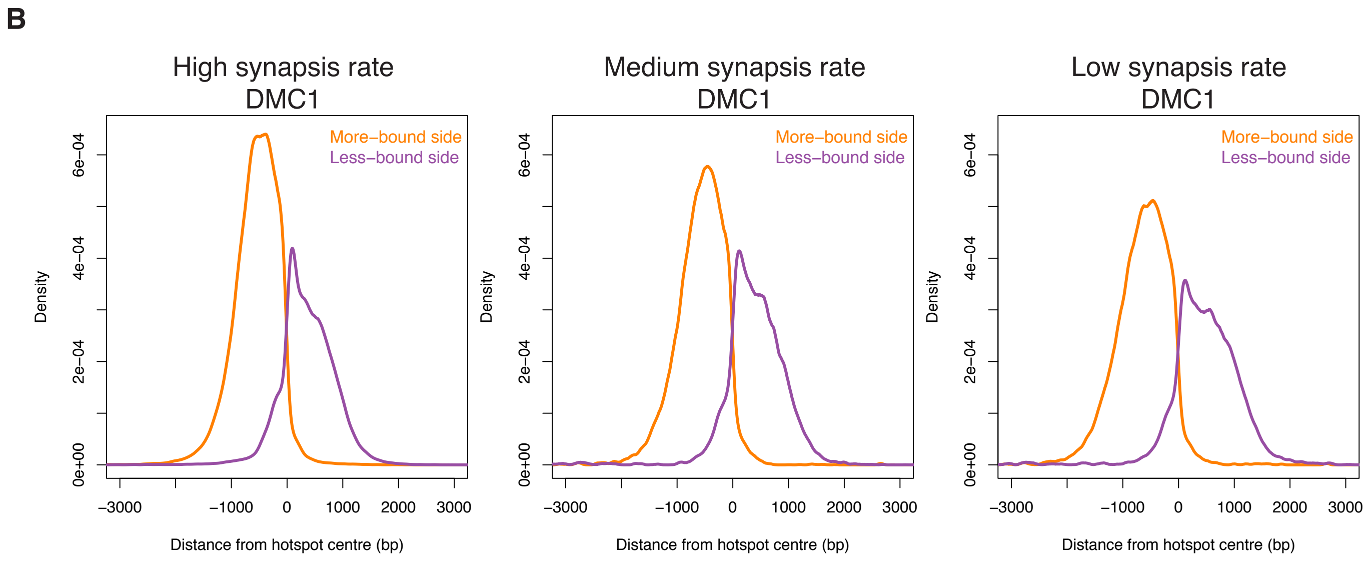

Figure S8 related to Figure 4: Imbalance in RPA, DMC1 and RAD51 binding may be due to differences in the efficiency of break processing on the two sides of a break in skewed hotspots

(A) Comparison of RPA on the DSB-initiating (light blue, above the x-axis) and the repair-template (dark blue, below the x-axis) chromosomes in hotspots with skewed DMC1. Hotspots were co-oriented such that the more-bound side was to the left (left plot) and the less-bound side was to the right (right plot). The side with more binding of RPA on the DSB-initiating chromosome also has more binding of RPA on the repair-template chromosome.
(B) Comparison of the DMC1 signal in a set of skewed PRDM9^{B6} hotspots in three different mouse strains, all of which have a PRDM9^{B6} allele but have different speeds and success rates of homologue engagement and synapsis. These strains are B6 wild-type (left), B6xPWD (middle, data from (Davies et al., 2016)), and PWDxB6 (data from (Davies et al., 2016)), of which B6 is fertile, B6xPWD is sub-fertile and PWDxB6 is completely infertile due to failure to synapse chromosomes and repair breaks in meiotic prophase I. To assess whether the skew in protein in binding in B6 hotspots could reflect a difference in the dynamic behaviour of the two sides flanking a break, we compared the same set of hotspots (inferred from the B6 wild-type) with the other two strains. The skew is greatest in B6 and smallest in PWDxB6, which suggests a convergence of the shapes and intensities of protein binding on the two sides of a break as it continues to remain unrepaired. Note that the same set of hotspot locations is shown for each mouse, and therefore the underlying reference DNA sequence in these instances is the same. This rules out artefacts of mapping of sequencing reads as an explanation for the skew.
(C) As panel **A** but for DMC1 (top) and RAD51 (bottom). We fit linear models (see also Fig. 4A, S6A) to the shape of RPA binding on the more- and less-bound sides of the DSB-initiating chromosome using the shapes of DMC1 and RAD51 binding on the respective sides. The best-fitting linear models have similar proportions for both sides (more bound side: 65% DMC1 and 35% RAD51; less-bound side: 61% DMC1, 39% RAD51).

Supplementary Figure S9

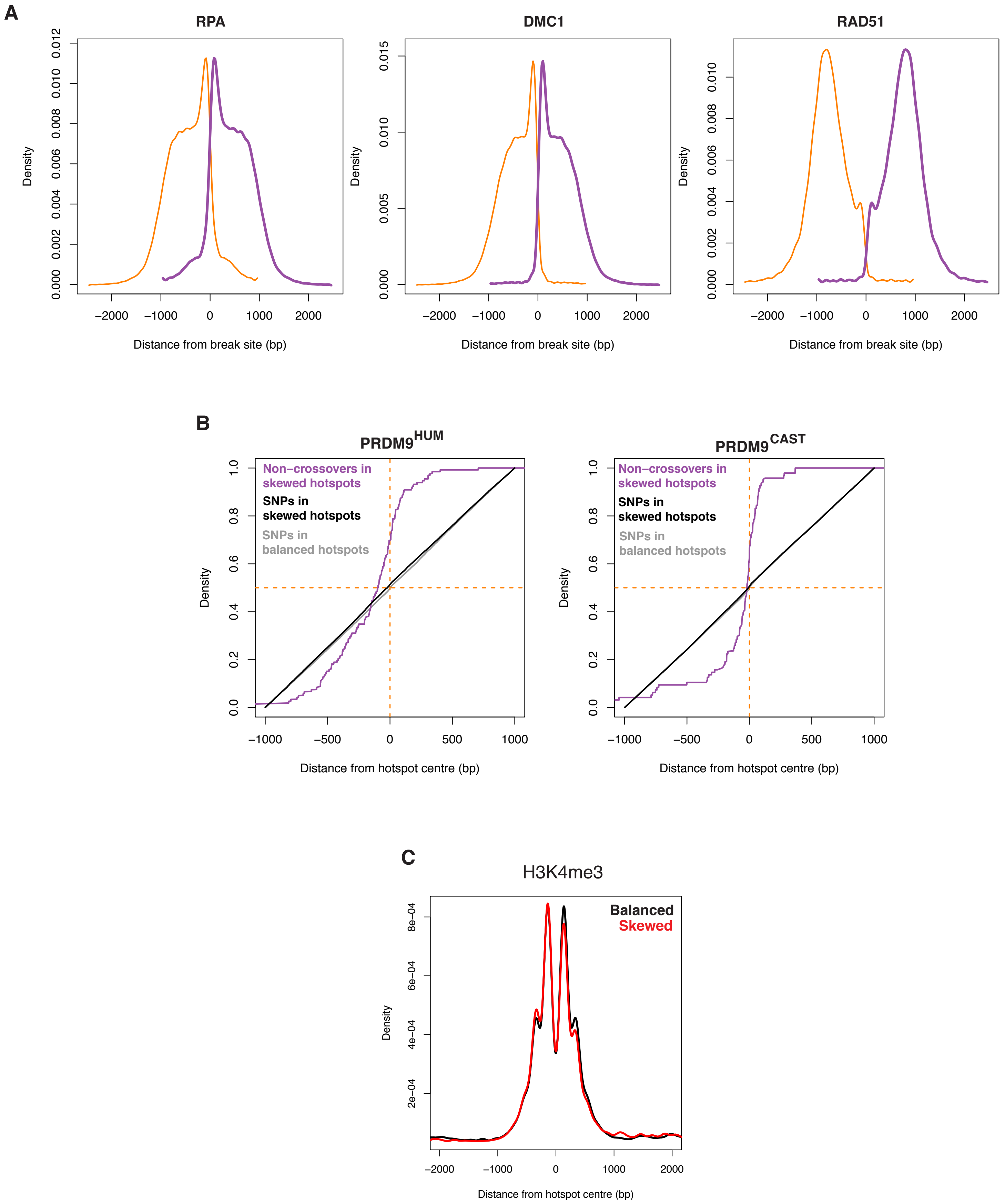

Figure S9 related to Figure 4: Comparisons of protein binding, non-crossover localization, SNP density and the H3K4me3 mark in skewed and balanced hotspots.

(A) Comparison of binding of RPA (left), DMC1 (middle), and RAD51 (right), on the two sides of a break in balanced hotspots in the wild-type B6 mouse.
(B) Assessing whether artefactual differences in SNP density may explain the shift in non-crossovers observed in skewed hotspots. (Left) Comparison of the cumulative distribution function of non-crossover tracts in skewed $PRDM9^{HUM}$ hotspots (purple) with that of SNPs in those hotspots (black) and balanced $PRDM9^{HUM}$ hotspots (grey) in the hybrid. The skew in non-crossover localisation cannot be explained by SNP density. (Right) As (left) but for $PRDM9^{CAST}$ hotspots.
(C) Comparison of the signal of H3K4me3 (data from (Baker et al., 2014)) in skewed (red) and balanced (black) hotspots in the B6 wild-type mouse.

Supplementary Figure S10

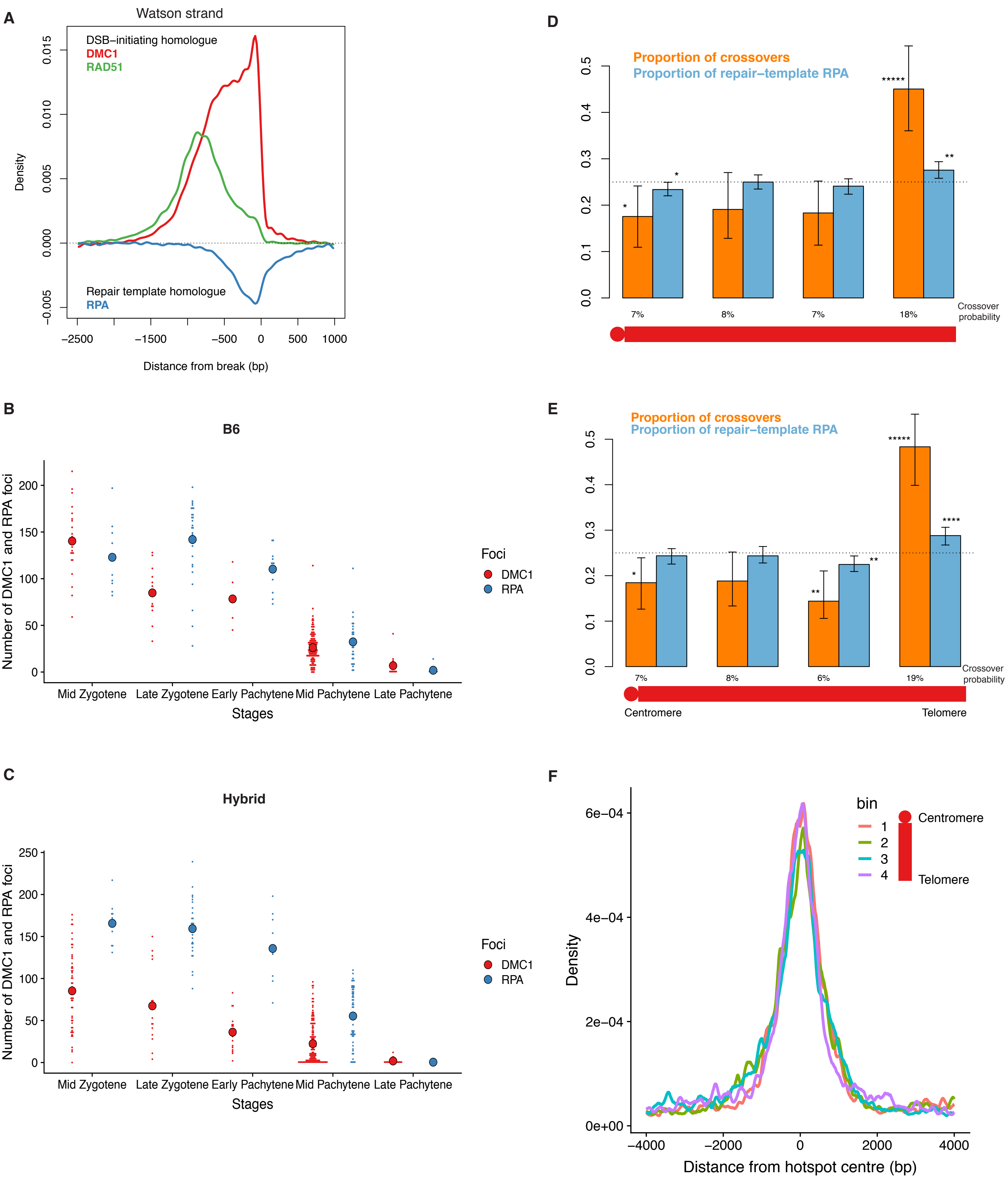

Figure S10 related to Figure 5: Cytogenetics of RPA foci and the intensity and localisation of RPA on the repair-template chromosome

(A) As Fig. 5A, but for the Watson strand.
(B) Comparison of the number of DMC1 (red) and RPA (blue) foci in several stages of meiotic prophase I in the B6 wild-type mouse. Each small dot shows the number of foci counted in one cell, with the large dots indicating the mean number of foci per stage per protein (number of cells: n=180 for DMC1, n=91 for RPA). The large numbers of RPA foci (>100 on average in early pachytene) imply that they cannot all be resolved as crossovers (~24 per male meiosis), and many if not most are resolved as non-crossovers, in agreement with previous work (Froenicke et al., 2002; Gasior et al., 1998; Hinch et al., 2019; Liu et al., 2014; Moens et al., 2002; Oliver-Bonet et al., 2005; Woglar and Villeneuve, 2018).
(C) As panel **B** but for the hybrid mouse (number of cells: n=261 for DMC1, n=113 for RPA).
(D) In Fig. 5C, we used a larger set of hotspots to gain power, with on average ~10X difference in recombination initiation between the homologues. Here we show the same analysis for the more stringent set of asymmetric hotspots that have ~20-fold more DSB-initiation on one homologue than the other on average (n=2,426).
(E) As Fig. 5C, but binning hotspots such that the total H3K4me3 signal on the DSB-initiating chromosome is the same for all bins (as opposed to RPA on the DSB-initiating chromosome used in Fig. 5C).
(F) RPA on the repair template chromosome in asymmetric $PRDM9^{CAST}$ hotspots in bins corresponding to Fig. S5D (sum of Watson and Crick strands, 100 bp smoothing). The estimated crossover resolution probability shows almost 3-fold variation amongst these bins. The proportion of repair-template RPA signal that is due to crossovers in each of these bins is approximately 16%, 17%, 16% and 45% respectively on average (after accounting for difference in lifespans and/or amounts). The localisation of RPA on the repair-template chromosome appears to be similar in different chromosomal regions.

Supplementary Table S1

	More-bound side	Less-bound side
PRDM9^{HUM} hotspots	92	40
PRDM9^{CAST} hotspots	59	35
Total	151	75

Table S1: (related to Figure 4) Non-crossovers in skewed hotspots. The number of non-crossovers for which the mid-point of the gene-conversion tract overlaps the more-bound and less-bound sides respectively in PRDM9^{HUM} and PRDM9^{CAST} hotspots (non-crossover location data are from Li et al., 2019). Only non-crossovers within 2 kb of hotspots with a unique well-defined PRDM9 motif were included. Hotspots that were within 8 kb of another hotspot were also excluded. Complex non-crossovers were excluded and data from all generations were pooled.

Supplementary Table S2

	More-bound side	Less-bound side
$PRDM9^{HUM}$ (pedigree)	114	71
$PRDM9^{HUM}$ (single-sperm)	33	35
$PRDM9^{CAST}$ (pedigree)	63	36
$PRDM9^{CAST}$ (single-sperm)	75	59
Total	285	201

Table S2: (related to Figure 4) Crossovers in skewed hotspots. Crossover break-points are identified using two informative single-nucleotide polymorphisms, each of which maps to a different parental haplotype. There is therefore a region of uncertainty within which the true crossover break-point occurs. Here we show the number of crossovers for which the mid-point of this "break-point region" overlaps the more-bound and less-bound sides respectively in $PRDM9^{HUM}$ and $PRDM9^{CAST}$ hotspots. Breakpoint location data are from Li et al., 2019 and Hinch et al., 2019 (pedigree and single-sperm, respectively). Only crossover break-points overlapping hotspots with a unique well-defined PRDM9 motif, without another hotspot within 8 kb, and with a region of uncertainty ≤ 1 kb were included, complex crossovers were excluded and data from all generations were pooled.

The smaller effect size for crossovers may reflect the fact that crossover break-points are not as well localised as non-crossovers. In addition, we are able to recover only one of the two break-points for a crossover gene-conversion tract. There is less power to detect a true effect with crossovers as it is not possible to identify, in general, which of the two ends of a DSB a particular crossover break-point is associated with.

Supplementary Table S3

	Coefficient	P-value
H3K4me3 (DSB-initiating chromosome)	+0.02	0.06
H3K4me3 (repair-template chromosome)	-0.01	0.34
RPA (DSB-initiating chromosome)	+0.02	0.34
RPA (repair-template chromosome)	+0.10	$\mathbf{10^{-21}}$
DMC1 (DSB-initiating chromosome)	+0.01	0.81
DMC1 (repair-template chromosome)	-0.02	0.50
RAD51 (DSB-initiating chromosome)	-0.04	0.11
RAD51 (repair-template chromosome)	0	0.94

Table S3: (related to Figure 5) Full linear model for predicting crossover outcomes from measures of DSB-initiation and repair in asymmetric hotspots in the hybrid: H3K4me3, RPA, DMC1, and RAD51 on each homologue.

Supplementary Table S4

	Coefficient	P-value
RPA on the repair-template chromosome	+0.09	$\mathbf{4 \times 10^{-40}}$
H3K4me3 on the DSB-initiating chromosome	+0.01	0.10

Table S4: (related to Figure 5) "Optimal" linear model for predicting crossover outcomes from measures of DSB-initiation and repair: H3K4me3, RPA, DMC1, and RAD51 on each homologue inferred using stepwise regression employing the Aikake Information Criterion (AIC) [Hastie and Pregibon, 1992], a standard method for variable selection. The full model is shown in Table S3.
